# Novel coumarin-6-sulfonamides as apoptotic anti-proliferative agents: synthesis, *in vitro* biological evaluation, and QSAR studies

**DOI:** 10.1080/14756366.2018.1477137

**Published:** 2018-06-26

**Authors:** Ahmed Sabt, Omaima M. Abdelhafez, Radwan S. El-Haggar, Hassan M. F. Madkour, Wagdy M. Eldehna, Ezz El-Din A. M. El-Khrisy, Mohamed A. Abdel-Rahman, Laila. A. Rashed

**Affiliations:** a Chemistry of Natural Compounds Department, National Research Centre, Dokki, Egypt;; b Pharmaceutical Chemistry Department, Faculty of Pharmacy, Helwan University, Cairo, Egypt;; c Chemistry Department, Faculty of Science, Ain-shams University, Cairo, Egypt;; d Department of Pharmaceutical Chemistry, Faculty of Pharmacy, Kafrelsheikh University, Kafrelsheikh, Egypt;; e Department of Pharmaceutical Chemistry, Faculty of Pharmacy, Egyptian Russian University, Badr City, Cairo, Egypt;; f Department of Biochemistry, Faculty of Medicine, Cairo University, Cairo, Egypt

**Keywords:** Anticancer, Apoptosis, Coumarin-6-sulfonamides, 2D-QSAR, Synthesis

## Abstract

Herein, we report the synthesis of different novel sets of coumarin-6-sulfonamide derivatives bearing different functionalities (**4a, b**, **8a–d**, **11a–d, 13a, b,** and **15a–c**), and *in vitro* evaluation of their growth inhibitory activity towards the proliferation of three cancer cell lines; HepG2 (hepatocellular carcinoma), MCF-7 (breast cancer), and Caco-2 (colon cancer). HepG2 cells were the most sensitive cells to the influence of the target coumarins. Compounds **13a** and **15a** emerged as the most active members against HepG2 cells (IC_50_ = 3.48 ± 0.28 and 5.03 ± 0.39 µM, respectively). Compounds **13a** and **15a** were able to induce apoptosis in HepG2 cells, as assured by the upregulation of the Bax and downregulation of the Bcl-2, besides boosting caspase-3 levels. Besides, compound **13a** induced a significant increase in the percentage of cells at Pre-G1 by 6.4-folds, with concurrent significant arrest in the G2-M phase by 5.4-folds compared to control. Also, **13a** displayed significant increase in the percentage of annexin V-FITC positive apoptotic cells from 1.75–13.76%. Moreover, QSAR models were established to explore the structural requirements controlling the anti-proliferative activities.

## Introduction

Cancer is a very complex, widespread and lethal disease that affects approximately 14 million people every year. In 2018, the American Cancer Society predict that the new diagnosed cancer cases will reach to 1,735,350 and cancer death 595,690 cancer deaths in the United States^1^. Accordingly, the discovery of new anti-cancer agents with promising bioactivity and high therapeutic index is still an urgent need and a major challenge for researchers.

It is well established that natural and synthetic coumarin derivatives have attracted a great deal of interest due to their variety of biological and pharmacological properties, such as their anti-inflammatory^2^, antibacterial^3^, antiviral^4^, and anti-cancer activities^5^. In the current medical era, much attention has been extensively paid to modify and update coumarin-based drug leads from the point of view of drug design and medicinal chemistry to fulfill more effective and safe anti-cancer agents^6–8^.

**Figure 1.**
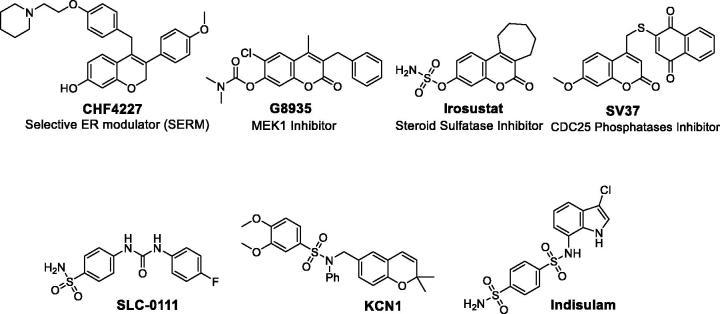
Structure of some reported coumarins and sulfonamides with effective anti-cancer activities.

The promising biological profile of coumarins as anti-cancer agents, and their easy synthetic modifications paved the way for design and synthesis of various coumarin-based derivatives with diverse activities against cancer. In this context, many coumarin-based molecules were widely reported to possess anti-cancer activity through binding to different targets and diverse pharmacological mechanisms, to name just a few, selective estrogen receptor modulation^9–11^ (as CHF4227, Figure 1), MEK1 inhibition^12–14^ (as G8935, Figure 1), steroid sulfatase inhibition^15–17^ (as Irosustat, Figure 1), CDC25 phosphatases inhibition^18–20^ (as SV37, Figure 1), tubulin polymerization inhibition^21–23^, aromatase inhibition^24^ ^,^ ^25^, COX-2 inhibition^26^, and apoptosis induction^27–^ ^31^.

On the other hand, sulfonamide derivatives constitute another important class of organic compounds that displayed interesting biological activities including anti-carbonic anhydrase and anti-cancer activities^32^ ^,^ ^33^. It is noteworthy to mention that there is a continuing interest in the synthesis of coumarin sulfonamide derivatives as potent anti-cancer agents^34–37^. SLC-0111, Figure 1, an ureido benzenesulfonamide derivative, is currently in phase I/II clinical trials as anti-cancer drug. SLC-0111 II is characterized by its selectivity towards inhibition of the transmembrane isoforms hCA IX/XII (over the cytosolic isoforms hCA I/II). SLC-0111 was also able to block human breast cancer invasion, delay tumor growth and diminish the cancer stem cell population *in vivo* ^38–40^.

KCN1, Figure 1, a novel synthetic sulfonamide, is a HIF pathway inhibitor with *in vitro* and *in vivo* anti-pancreatic cancer activities and preclinical pharmacology. KCN1 specifically inhibited HIF reporter gene activity in several glioma cell lines at the nanomolar level. Also, KCN1 effectively inhibited the growth of subcutaneous malignant glioma tumor xenografts with low side effects on the host^41^ ^,^ ^42^. Moreover, Indisulam (*N*-(-3-chloro-7-indolyl)-1,4-benzenedisulfonamide), Figure 1, is an orally active sulfonamide anti-tumor agent that possesses anti-cancer properties through down‐regulation of various cell‐cycle checkpoint molecules, thereby blocking the phosphorylation of retinoblastoma protein and inducing p53 and p21. Pre-clinical and clinical studies have established synergy of Indisulam with nucleoside analogs as well as topoisomerase inhibitors^43–47^.

Based on the aforementioned findings, herein we report the synthesis of novel sets of coumarin sulfonamide derivatives (**4a, b**, **8a–d**, **11a–d, 13a, b** and **15a–c**) and their *in vitro* growth inhibitory activity towards the proliferation of three cancer cell lines; HepG2 (hepatocellular carcinoma), MCF-7 (breast cancer), and Caco-2 (colon cancer). Additionally, the synthesized coumarin sulfonamides were further examined regarding their potential apoptotic induction and their effects on cell cycle progression in the Hep-G2 cancer cells to acquire a perception for the mechanism of their anti-cancer activity.

## Materials and methods

### Chemistry

Melting points were determined on Electrothermal IA 9000 apparatus and were uncorrected. Elemental microanalyses were performed on Elementar, Vario EL, at the Micro-analytical Laboratory, National Research Centre, Dokki, Cairo. The ^1^H NMR and ^13^C NMR spectra were recorded with a BrukerAvance 400 MHz spectrometer at Turku University, Finland and JEOL ACA 500 NMR spectrometer, at the National Research Centre, Dokki, Cairo, Egypt. The mass spectra were performed on Mass Spectrometer Finnigan MAT SSQ-7000 and GCMS-QP 1000EX Shimadzu Gas Chromatography MS Spectrometer at Faculty of Science, Cairo University, Egypt. The reactions were followed by TLC (silica gel, aluminum sheets 60 F254, Merck) using chloroform/methanol (9.5:0.5 v/v) as eluent.

### Synthesis of coumarin-6-sulfonyl chloride 2

Compound 2-oxo-2*H*-chromene-6-sulfonyl chloride **2** was obtained from a reaction of coumarin with chlorosulfonic acid^48^.

### Synthesis of coumarin-6-sulfonamide derivatives 4a,b

A solution of 2-oxo-2*H*-chromene-6-sulfonyl chloride **2** (0.5 g, 2 mmol) and the appropriate sulfanilamide **3a** or sulfapyridine **3b** (2 mmol) in absolute ethanol (25 ml) was refluxed for 12 h. The obtained solid was filtered, dried, and recrystallized from ethanol to give compounds **4a, b**.

### 2-Oxo-N-(4-sulfamoylphenyl)-2H-chromene-6-sulfonamide (4a)

Brown crystals, mp 266–268 °C, yield (43%). ^1^H NMR (500 MHz, DMSO-d6) *δ ppm*: 6.47 (d, H, *J* = 9.5 Hz, H3 of coumarin), 6.94 (d, 2H, *J* = 8.2 Hz, Ar), 7.31 (d, 1H, *J* = 8.5 Hz, H8 of coumarin), 7.61 (d, 2H, *J* = 8.4 Hz, Ar), 7.71–7.71 (m, 3H, NH_2_ and H7 of coumarin), 7.95 (d, 1H, *J* = 10 Hz, H4 of coumarin), 8.11 (s, 1H, H5 of coumarin), 10.95 (s, 1H, NH); ^13^C NMR (100 MHz, DMSO-d6); *δ ppm*: 116.32(2C), 116.90, 118.40, 118.84, 125.96, 125.97, 127.87 (2C), 129.81 (2C), 137.55, 144.94 (C4 of coumarin), 153.88 (C9 of coumarin), 160.40 (C=O of coumarin); MS m/z [%]: 380 [9.21], 92 [100]. Analysis for C_15_H_12_N_2_O_6_S_2_ (380), Calcd.: % C, 47.36; H, 3.18; N, 7.36; O, 25.24; S, 16.86. Found: % C, 47.28; H, 3.22; N, 7.42; O, 25.18; S, 16.78.

### 2-Oxo-N-(4-(N-(pyridin-2-yl)sulfamoyl)phenyl)-2H-chromene-6-sulfonamide (4b)

White crystals, mp 237–239 °C, yield (40%). ^1^H NMR (400 MHz, DMSO-d6) *δ ppm*: 6.62 (d, 1H, *J* = 9.6 Hz, H3 of coumarin), 6.83 (t, 1H, *J* = 12.4 Hz, H3 of pyridine), 7.08 (d, 1H, *J* = 8.8 Hz, H5 of pyridine), 7.24 (d, 2H, *J* = 8.8 Hz, Ar), 7.55 (d, 1H, *J* = 8.8 Hz, H8 of coumarin), 7.67 (t, 1H, *J* = 8.8 Hz, H4 of pyridine), 7.75 (d, 2H, *J* = 8.8 Hz, Ar), 7.94–7.96 (m, 2H, H2 of pyridine and H7 of coumarin), 8.15 (d, 1H, *J* = 10 Hz, H4 of coumarin), 8.28 (s, 1H, H5 of coumarin), 11.05 (s, 1H, NH, D_2_O exchangeable), 11.92 (s, 1H, NH, D_2_O exchangeable); ^13 ^C NMR (100 MHz, DMSO); *δ ppm*: 114.23 (C5 of pyridine), 118.42 (2C), 118.99 (2C), 119.49, 128.29, 128.66, 128.55, 129.88, 129.96, 135.38, 137.18, 140.90, 141.01, 141.29, (Ar, pyridine and coumarin), 143.76 (C4 of coumarin), 153.57 (C=N of pyridine), 156.51 (C9 of coumarin), 159.54 (C=O of coumarin); Analysis for C_20_H_15_N_3_O_6_S_2_ (457), Calcd.: % C, 52.51; H, 3.30; N, 9.19; O, 20.98; S, 14.02. Found: % C, 52.58; H, 3.40; N, 9.22; O, 21.01; S, 14.09.

### Synthesis of N-(4-acetylphenyl)-2-oxo-2H-chromene-6-sulfonamide (6)

Compound **6** was prepared according to the literatures procedures^49^.

### Synthesis of schiff base coumarin sulfonamides 8a-d

To a mixture of compound **6** (0.25 g, 0.729 mmol) and the appropriate phenylhydrazine **7a**, 2,4-dinitrophenylhydrazine **7b**, 2,4,6-trichlorophenylhydrazine **7c** or *p-*toluenesulfonyl hydrazide **7d** (0.911 mmol) in absolute ethanol (30 ml), three drops of glacial acetic acid was added and the reaction mixture was refluxed for 8 h. After cooling, the precipitate was filtered, washed with water, purified *via* crystallization from ethanol to give compounds **8a–d**, respectively.

### 2-Oxo-N-(4–(1-(2-phenylhydrazono)ethyl)phenyl)-2H-chromene-6-sulfonamide (8a)

Brown crystals, mp 178–180 °C, yield (66%). ^1^H NMR (500 MHz, DMSO-d6) *δ ppm*: 2.19 (s, 3H, CH_3_), 6.47 (d, 1H, *J* = 12 Hz, H3 of coumarin), 6.87–7.83 (m, 11H, NH, Ar, and H8 of coumarin), 7.95 (dd, 1H, *J* = 6 and 10.5 Hz, H7 of coumarin), 8.04 (d, 1H, *J* = 2.4 Hz, H4 of coumarin), 8.23 (s, 1H, H5 of coumarin), 11.09 (s, 1H, NH); ^13^C NMR (125 MHz, DMSO); *δ ppm*: 13.10 (CH_3_), 113.21 (2C), 113.25, 118.59, 119.25, 120.47, 122.66, 126.50, 128.18, 128.29, 129.32 (2C), 129.40, 130.33 (2C), 132.62, 135.93, 135.95, 143.81 (C4 of coumarin), 154.54 (C=N), 156.31 (C9 of coumarin), 160.02 (C=O of coumarin); HRMS (ESI): *m/z* calculated for C_23_H_19_N_3_O_4_S [M + H]^+^, 434.1169; found, 434.1163.

### N-(4–(1-(2–(2,4-dinitrophenyl)hydrazono)ethyl)phenyl)-2-oxo-2H-chromene-6-sulfonamide (8b)

Red crystals, mp 269–270 °C, yield (60%). ^1^H NMR (500 MHz, DMSO-d6) *δ ppm*: 2.36 (s, 3H, CH_3_), 5.72 (s, 1H, NH), 6.61 (d, 1H, *J* = 6.50 Hz, H3 of coumarin), 7.21(d, 2H, *J* = 11 Hz, Ar), 7.57–7.85 (m, 3H, Ar, and H8 of coumarin), 7.96 (dd, 1H, *J* = 2.5 and 2.5 Hz, H7 of coumarin), 8.03 (d, 1H, *J* = 12 Hz, H4 of coumarin), 8.19 (d, 1H, *J* = 12 Hz, Ar), 8.29 (s, 1H, H5 of coumarin), 8.36 (d, 1H, *J* = 12 Hz, NO_2_-CH, Ar), 8.87 (s, 1H, H3 of 2,4-(NO_2_)_2_C_6_H_3_), 11.06 (s, H, NH); ^13 ^C NMR (125 MHz, DMSO-d6); *δ ppm*: 13.83 (CH_3_), 118.26, 118.29, 118.38, 118.42, 118.60, 119.41, 119.50, 120.01, 127.26, 128.18, 128.29, 130.00, 130.05, 130.31, 132.60, 135.88, 138.33, 142.38, 143.86 (C4 of coumarin), 154.37 (C=N), 156.34 (C9 of coumarin), 159.56 (C=O of coumarin); Analysis for C_23_H_17_N_5_O_8_S (523), Calcd.: % C, 52.77; H, 3.27; N, 13.38; O, 24.45; S, 6.13. Found: % C, 52.84; H, 3.20; N, 13.32; O, 24.40; S, 6.18.

### 2-Oxo-N-(4–(1-(2–(2,4,6-trichlorophenyl)hydrazono)ethyl)phenyl)-2H-chromene-6-sulfonamide (8c)

White crystals, mp 259–260 °C, yield (58%). ^1^H NMR (500 MHz, DMSO-d6) *δ ppm*: 2.21 (s, 3H, CH_3_), 6.55 (d, 1H, *J* = 12 Hz, H3 of coumarin), 7.11 (d, 2H, *J* = 10.5 Hz, Ar), 7.54–7.60 (m, 3H, Ar, and H8 of coumarin), 7.64 (s, 2H, H3 and H5 of 2,4,6-(Cl)_3_C_6_H_2_), 7.92 (dd, 1H, *J* = 2 and 2 Hz, H7 of coumarin), 8.04 (s, 1H, NH), 8.16 (d, 1H, *J* = 12.5 Hz, H4 of coumarin), 8.22 (s, 1H, H5 of coumarin), 10.51 (s, 1H, NH); ^13 ^C NMR (125 MHz, DMSO-d6); *δ ppm*: 12.98 (CH_3_), 118.23, 118.29, 118.36, 119.39, 120.21 (2C), 126.78 (2C), 128.06, 128.17, 128.86, 129.14 (2C), 130.03, 134.92, 135.86, 137.80, 139.14, 143.85 (C4 of coumarin), 145.64 (C=N), 156.33 (C9 of coumarin), 159.54 (C=O of coumarin); Analysis for C_23_H_16_Cl_3_N_3_O_4_S (534), Calcd.: % C, 51.46; H, 3.00; Cl, 19.81; N, 7.83; O, 11.92; S, 5.97. Found: % C, 51.49; H, 2.96; Cl, 19.85; N, 7.85 O, 11.87; S, 5.92.

### 2-Oxo-N-(4–(1-(2-tosylhydrazono)ethyl)phenyl)-2H-chromene-6-sulfonamide (8d)

White crystals, mp 163–165 °C, yield (76%). ^1^H NMR (400 MHz, DMSO-d6) *δ ppm*: 2.03 (s, 3H, CH_3_), 2.31 (s, 3H, CH_3_ - tosyl), 6.60 (d, 1H, *J* = 9.6 Hz, H3 of coumarin), 7.11 (d, 2H, *J* = 8.4 Hz, Ar), 7.39 (d, 2H, *J* = 8.4 Hz, Ar-tosyl), 7.50–7.56 (m, 3H, Ar, and H8 of coumarin), 7.78 (d, 2H, *J* = 8.4 Hz, Ar-tosyl), 7.91 (dd, 1H, *J* = 2 and 2 Hz, H7 of coumarin), 8.17 (d, 1H, *J* = 9.6 Hz, H4 of coumarin), 8.18 (s, 1H, H5 of coumarin), 10.44 (s, 1H, NH), 10.56 (s, 1H, NH); ^13 ^C NMR (100 MHz, DMSO); *δ ppm*: 14.47 (CH_3_), 21.47 (CH_3_), 118.30, 118.45, 118.53, 119.42, 119.75, 125,77, 127.50, 128.04 (2C), 128.18, 129.91, 129.97 (2C), 133.45, 135.70, 136.57, 138.90, 143.79, 143.83 (C4 of coumarin), 152.92 (C=N), 156.37 (C9 of coumarin), 159.56 (C=O of coumarin); Analysis for C_24_H_21_N_3_O_6_S_2_ (511), Calcd.: % C, 56.35; H, 4.14; N, 8.21; O, 18.77; S, 12.54. Found: % C, 56.40; H, 4.08; N, 8.16; O, 18.71; S, 12.50.

### 2–(1-(4–(2-Oxo-2H-chromene-6-sulfonamido)phenyl)ethylidene)hydrazine carbothioamide (9)

A mixture of *N*-(4-acetylphenyl)-2-oxo-2*H*-chromene-6-sulfonamide **6** (0.5 g, 1.45 mmol), and thiosemicarbazide (1.45 mmol) in absolute ethanol (20 ml) and catalytic amount of acetic acid was refluxed for 6 h. The obtained solid was filtered, dried, and washed with hot ethanol to afford compound **9**.

Light yellow crystals, mp 253–254 °C, yield (34%). ^1^H NMR (400 MHz, DMSO-d6) *δ ppm*: 2.18 (s, 3H, CH_3_), 6.58 (d, 1H, *J* = 4.4 Hz, H3 of coumarin), 7.25 (d, 2H, *J* = 6.8 Hz, Ar), 7.55 (d, 1H, *J* = 2.4 Hz, H8 of coumarin), 7.79–7.84 (m, 4H, NH_2_, Ar), 7.94 (dd, 1H, *J* = 6 and 1.6 Hz, H7 of coumarin), 8.16 (d, 1H, *J* = 10 Hz, H4 of coumarin), 8.29 (d, 1H, *J* = 2.4 Hz, H5 of coumarin), 10.13 (s, 1H, NH), 10.60 (s, 1H, NH); ^13 ^C NMR (100 MHz, DMSO-d6); *δ ppm*: 14.17 (CH_3_), 113.57, 118.30, 118.34, 119.43 (2C), 119.69, 128.14 (2C), 130.01, 133.82, 135.86, 138.72, 143.82 (C4 of coumarin), 147.53 (C=N), 156.36 (C9 of coumarin), 159.54 (C=O of coumarin), 179.22 (C = S), Analysis for C_18_H_16_N_4_O_4_S_2_ (416), Calcd.: % C, 51.91; H, 3.87; N, 13.45; O, 15.37; S, 15.40. Found: %C, 51.79; H, 3.82; N, 13.31; O, 15.42; S, 15.47.

### General procedures for preparation of target compounds (**11a–d** and **13a**, **b**)

A mixture of thiosemicarbazone **9** (10 mmol) and the appropriate α-halocarbonyl compounds **10a–d**, phenacyl bromide **12a** or coumarin-3-acetylbromide **12 b** (10 mmol) in dioxane (25 ml) containing catalytic amount of triethylamine was heated under reflux for 8 h and then cooled. The solution was poured onto water-ice and concentrated hydrochloric acid. The solid produced was collected by filtration and crystallized from ethanol to furnish compounds **11a–d** and **13a**, **b,** respectively.

### N-(4–(1-(2–(4-methylthiazol-2-yl)hydrazono)ethyl)phenyl)-2-oxo-2H-chromene-6-sulfonamide (11a)

Brown crystals, mp 257–259 °C, yield (78%). ^1^H NMR (500 MHz, DMSO-d6) *δ* = 2.15 (s, 3H, CH_3_), 2.45 (s, 3H, CH_3_-thiazole), 5.99 (s, 1H, CH thiazole), 6.57 (d, 1H, *J* = 9.6 Hz, H3 of coumarin), 7.19 (d, 2H, *J* = 8.6 Hz, Ar), 7.52 (d, 1H, *J* = 8.6 Hz, H8 of coumarin), 7.80 (d, 2H, *J* = 8.6 Hz, Ar), 7.91 (dd, 1H, *J* = 9.5 and 2 Hz, H7 of coumarin), 8.12 (d, 1H, *J* = 10 Hz, H4 of coumarin), 8.27 (s, 1H, H5 of coumarin), 10.55 (s, 1H, NH, D_2_O exchangeable), 11.01 (s, 1H, NH, D_2_O exchangeable); ^13 ^C NMR (125 MHz, DMSO); *δ ppm*: 13.83 (CH_3_), 14.55 (CH_3_-thiazole), 118.26, 118.42, 118.60, 119.41, 119.50, 120.01, 127.26, 128.18, 130.31, 132.60, 135.88, 138.33, 142.38 (Ar, C5-thiazole and coumarin), 143.86 (C4 of coumarin), 154.33 (C=N), 154.37 (C4-thiazole), 156.34 (C9 of coumarin), 159.56 (C=O of coumarin), 168.66 (S-C=N): Analysis for C_21_H_18_N_4_O_4_S_2_ (454), Calcd.: % C, 55.49; H, 3.99; N, 12.33; O, 14.08; S, 14.11. Found: % C, 55.40; H, 3.96; N, 12.36; O, 14.05; S, 14.14.

### Ethyl 4-methyl-2–(2-(1–(4-(2-oxo-2H-chromene-6-sulfonamido)phenyl) ethylidene)hydrazinyl)thiazole-5-carboxylate (11b)

Yellow crystals, mp 180–182 °C, yield (53%). ^1^H NMR (500 MHz, DMSO-d6) *δ ppm*: 1.21 (t, 3H, *J* = 11.48 Hz, CH_3_), 2.19 (s, 3H, CH_3_), 2.41 (s, 3H, CH_3_-thiazole), 4.14 (q, 2H, *J* = 6.65 Hz, OCH_2_), 6.56 (d, 1H, *J* = 9.2 Hz, H3 of coumarin), 7.14 (d, 2H, *J* = 8.4 Hz, Ar), 7.53 (d, 1H, *J* = 8.6 Hz, H8 of coumarin), 7.63 (d, 2H, *J* = 8.4 Hz, Ar), 7.91 (dd, 1H, *J* = 9.6 and 5 Hz, H7 of coumarin), 8.14 (d, 1H, *J* = 9.6 Hz, H4 of coumarin), 8.22 (s, 1H, H5 of coumarin), 10.67 (s, 1H, NH), 11.04 (s, 1H, NH); ^13 ^C NMR (125 MHz, DMSO-d6); *δ ppm*: 14.62 (CH_3_), 14.87 (CH_3_), 26.93 (CH_3_-ester), 60.69 (OCH_2_), 112.87, 118.34, 118.67, 119.48, 120.01, 127.51, 128.25, 130.13 (2C), 130.37, 135.91 (Ar, C5-thiazole and coumarin), 143.87 (C4 of coumarin), 152.41 (C=N), 156.41 (C4-thiazole), 156.53 (C9 of coumarin), 159.48 (C=O of coumarin), 169.66 (S-C=N), 185.45 (C=O); HRMS (ESI): *m/z* calculated for C_24_H_22_N_4_O_4_S_2_ [M + H]^+^, 527.1054; found, 527.1052.

### N-(4–(1-(2–(5-((4-chlorophenyl)diazenyl)-4-methylthiazol-2-yl)hydrazono)ethyl) phenyl)-2-oxo-2H-chromene-6-sulfonamide (11c)

Red crystals, mp 250–252 °C, yield (88%). ^1^H NMR (500 MHz, DMSO-d6) *δ ppm*: 2.41 (s, 3H, CH_3_), 2.57(s, 3H, CH_3_-thiazole), 6.62 (d, 1H, *J* = 12 Hz, H3 of coumarin), 7.21 (d, 2H, *J* = 10.5 Hz, Ar), 7.35–7.39 (m, 4H, Ar), 7.59 (d, 1H, *J* = 8.6 Hz, H8 of coumarin), 7.85 (d, 2H, *J* = 8.8 Hz, Ar), 7.97 (dd, 1H, *J* = 3 and 3 Hz, H7 of coumarin), 8.19 (d, 1H, *J* = 12 Hz, H4 of coumarin); 8.30 (s, 1H, H5 of coumarin), 10.65 (br, 2H, 2NH,); ^13 ^C NMR (125 MHz, DMSO) *δ ppm*: 14.55 (CH_3_), 15.50 (CH_3_), 118.35, 118.38, 118.64, 119.47 (2C), 119.98, 123.52, 124.53, 128.22, 128.54, 129.64 (2C), 129.99, 130.04, 130.31, 133.07, 135.92, 139.30, 140.32 (Ar, C5-thiazole and coumarin), 143.84 (C4 of coumarin), 147.89 (C4-thiazole), 151.37 (C=N), 156.42 (C9 of coumarin), 159.54 (C=O of coumarin), 171.80 (S-C=N); Analysis for C_27_H_21_ClN_6_O_4_S_2_ (592), Calcd.: % C, 54.68; H, 3.57; Cl, 5.98; N, 14.17; O, 10.79; S, 10.81. Found: % C, 54.61; H, 3.52; N, 14.20; O, 10.71; S, 10.70.

### N-(4–(1-(2–(4-methyl-5-(p-tolyldiazenyl)thiazol-2yl)hydrazono)ethyl)phenyl)-2-oxo-2H-chromene-6-sulfonamide (11d)

Red crystals, mp 258–259 °C, yield (83%). ^1^H NMR (500 MHz, DMSO-d6) *δ ppm*: 2.25 (s, 3H, CH_3_), 2.41 (s, 3H, CH_3_), 2.56 (s, 3H, CH_3_-thiazole), 6.62 (d, 1H, *J* = 12 Hz, H3 of coumarin), 7.13 (d, 2H, *J* = 10 Hz, Ar), 7.20–7.25 (m, 4H, Ar), 7.59 (d, 1H, *J* = 11 Hz, H8 of coumarin), 7.85 (d, 2H, *J* = 10.5 Hz, Ar), 7.97 (dd, 1H, *J* = 3 and 3 Hz, H7 of coumarin), 8.19 (d, 1H, *J* = 12 Hz, H4 of coumarin), 8.29 (s, 1H, H5 of coumarin), 10.47 (s, 1H, NH), 10.77 (s, 1H, NH); ^13 ^C NMR (125 MHz, DMSO); *δ ppm*: 15.48 (CH_3_), 16.94 (CH_3_), 20.85 (CH_3_), 114.68, 118.39, 118.60, 119.47 (2C), 128.23, 128.28, 128.52, 129.99, 130.04, 130.20, 130.33, 131.65, 133.26, 135.63, 135.83, 137.96, 140.10, 141.61, 142.35 (Ar, thiazole and coumarin), 143.83 (C4 of coumarin), 151.15 (C=N), 156.44 (C9 of coumarin), 159.54 (C=O of coumarin), 171.79 (S-C=N); Analysis for C_28_H_24_N_6_O_4_S_2_ (572), Calcd.: % C, 58.73; H, 4.22; N, 14.68; O, 11.18; S, 11.20. Found: % C, 58.82; H, 4.14; N, 14.77; O, 11.19; S, 11.16.

### 2-Oxo-N-(4–(1-(2–(4-phenylthiazol-2-yl)hydrazono)ethyl)phenyl)-2H-chromene-6-sulfonamide (13a)

Yellow crystals, mp 170–171 °C, yield (80%). ^1^H NMR (500 MHz, DMSO-d6) *δ ppm*: 2.19 (3H, s, CH_3_), 6.56 (d, 1H, *J* = 5.75 Hz, H3 of coumarin), 7.16 (d, 2H, *J* = 8.8 Hz, Ar), 7.27–7.36 (m, 5H, Ar), 7.54 (d, 1H, *J* = 8.65 Hz, H8 of coumarin), 7.61 (d, 2H, *J* = 8.4 Hz, Ar), 7.94 (dd, 1H, *J* = 2.4 and 8 Hz, H7 of coumarin), 8.15(d, 1H, *J* = 8 Hz, H4 of coumarin), 8.20 (s, 1H-thiazole), 8.29 (d, 1H, *J* = 2.5 Hz, H5 of coumarin), 10.59 (s, 1H, NH), 10.99 (s, 1H, NH); ^13^C NMR (125 MHz, DMSO-d6); *δ ppm*: 14.29 (CH_3_), 104.44 (C4-thiazole), 118.29, 118.58, 119.43, 120.11, 125.96, 128.20, 128.29, 129.06 (2C), 130.05, 130.33, 132.61, 134.20, 135.21, 135.61, 135.86, 138.26, 142.35 (Ar, and coumarin), 143.86 (C4 of coumarin), 146.27 (C5-thiazole), 156.36 (C=N), 156.50 (C9 of coumarin), 159.56 (C=O of coumarin), 170.21 (S-C=N);. MS *m/z* [%]: 516 [93], 132 [100]; Analysis for C_26_H_20_N_4_O_4_S_2_ (516), Calcd.: % C, 60.45; H, 3.90; N, 10.85; O, 12.39; S, 12.41 Found: % C, 60.39; H, 3.88; N, 10.87; O, 12.33; S, 12.36.

### 2-Oxo-N-(4–(1-(2–(4-(2-oxo-2H-chromen-3-yl)thiazol-2-yl)hydrazono)ethyl)phenyl)-2H-chromene-6-sulfonamide (13b)

Yellow crystals, mp 201–202 °C, yield (63%). ^1^H NMR (500 MHz, DMSO-d6) *δ ppm*: 2.16 (s, 3H, CH_3_), 6.58 (d, 1H, *J* = 9.3 Hz, H3 of coumarin), 7.20 (d, 2H, *J* = 8.1 Hz, Ar), 7.34–8.80 (m, 7H, 8H coumarin and Ar), 7.97 (dd, 1H, *J* = 8.6 and 9.5 Hz, H7 of coumarin), 8.11 (s, 1H, H4 of coumarin), 8.15 (d, 1H, *J* = 9.5 Hz, H4 of coumarin), 8.28 (d, 1H, *J* = 8.6 Hz, H5 of coumarin), 8.52 (s, 1H-thiazole), 10.60 (s, 1H, NH); 10.99 (s, 1H, NH); ^13 ^C NMR (125 MHz, DMSO-d6); *δ ppm*: 14.32 (CH_3_), 111.46 (C4-thiazole), 116.36, 118.30, 118.43, 118.58 (2C), 119.50, 120.05, 125.20, 127.17, 128.29, 129.20 (2C), 129.98, 130.33 (2C), 132.61, 134.03, 135.61, 135.84, 142.35, 143.80 (C4 of coumarin), 152.75 (C=N), 156.36 (C5-thiazole), 156.50 (2C9 of coumarin), 159.51 (2C=O of coumarin), 169.67 (S-C=N); Analysis for C_29_H_20_N_4_O_6_S_2_ (584), Calcd.: % C, 59.58; H, 3.45; N, 9.58; O, 16.42; S, 10.97. Found: % C, 59.52; H, 3.48; N, 9.56; O, 16.36; S, 10.94.

### General procedures for synthesis of thiazolidinones (15a–c)

In 50 ml round-bottom flask, thiosemicarbazone **9** (10 mmol) was dissolved in 15 ml acetic acid, followed by addition of anhydrous sodium acetate (30 mmol) under magnetic stirring and warming. After 20 min, bromoacetic acid **14a**, 2-bromopropanoic acid **14b** or maleic anhydride **14c** (15 mmol) was added and the reaction mixture was maintained under reflux for 8 h. After cooling, the precipitate was filtered, washed with water, dried, and recrystallized from ethanol to give compounds **15a–c,** respectively.

### 2-Oxo-N-(4–(1-(2–(5-oxo-4,5-dihydrothiazol-2-yl)hydrazono)ethyl)phenyl)-2H-chromene-6-sulfonamide (15a)

Yellow crystals, mp 191–192 °C, yield (64%). ^1^H NMR (400 MHz, CDCl_3_) *δ ppm*: 2.23 (s, 3H, CH_3_), 3.8 (s, 2H, CH_2_, thiazolidinone), 6.62 (d, 1H, *J* = 10.8 Hz, H3 of coumarin), 7.25 (d, 2H, *J* = 12 Hz, Ar), 7.55 (d, 1H, *J* = 8.4 Hz, H8 of coumarin); 7.85 (d, 2H, *J* = 8.4 Hz, Ar), 7.95 (dd, 1H, *J* = 2.4 and 2 Hz, H7 of coumarin), 8.16 (d, 1H, *J* = 10 Hz, H4 of coumarin), 8.29 (s, 1H, H5 of coumarin), 10.66 (s, 1H, NH), 10.97 (s, 1H, NH); ^13 ^C NMR (100 MHz, CDCl_3_); *δ ppm*: 15.33 (CH_3_), 56.30 (CH_2_), 118.39, 118.64 (2C), 119.72, 127.93, 128.26, 129.99 (2C), 130.30, 132.66, 135.87, 142.35, 143.79 (C4 of coumarin), 152.56 (C=N), 156.50 (C9 of coumarin), 159.48 (C=O of coumarin), 166.33 (S-C=N), 196.90 (C=O); Analysis for C_20_H_16_N_4_O_5_S_2_ (456), Calcd.: % C, 52.62; H, 3.53; N, 12.27; O, 17.52; S, 14.05. Found: % C, 52.58; H, 3.50; N, 12.19; O, 17.50; S, 14.00.

### N-(4–(1-(2–(4-methyl-5-oxo-4,5-dihydrothiazol-2-yl)hydrazono)ethyl)phenyl)-2-oxo-2H-chromene-6-sulfonamide (15b)

Brown crystals, mp 180–182 °C, yield (50%). ^1^H NMR (400 MHz, CDCl_3_) *δ ppm*: 1.59 (d, 3H, *J* = 9 Hz, CH_3_ thiazolidinone), 2.27 (s, 3H, CH_3_), 3.96 (q, 1H, *J* = 9 Hz CH thiazolidinone), 5.23 (s, 1H, NH), 6.44 (d, 1H, *J* = 10 Hz, H3 of coumarin), 7.05 (d, 2H, *J* = 10.5 Hz, Ar), 7.30 (d, 1H, *J* = 10.5 Hz, H8 of coumarin), 7.61 (d, 1H, *J* = 12 Hz, H4 of coumarin), 7.69 (d, 2H, *J* = 10.5 Hz, Ar), 7.84 (dd, 1H, *J* = 5.5 and 3 Hz, H7 of coumarin), 7.95 (s, 1H, H5 of coumarin), 11.02 (s, 1H, NH); ^13 ^C NMR (100 MHz, CDCl_3_); *δ ppm*: 13.70 (CH_3_), 18.17 (CH_3_), 41.47 (CH of thiazolidinone), 117.10, 117.49, 117.88, 119.85 (2C), 126.66, 127.03 (2C), 129.05, 134.25, 136.40, 140.69, 141.30 (C4 of coumarin), 148.51 (C=N), 155.60 (C9 of coumarin); 158.13 (C=O of coumarin), 172.21 (S-C=N), 180.55 (C=O); Analysis for C_21_H_18_N_4_O_5_S_2_ (470), Calcd.: % C, 53.61; H, 3.86; N, 11.91; O, 17.00; S, 13.63. Found: % C, 53.55; H, 3.83; N, 11.87; O, 16.98; S, 13.60.

### 2–(4-Oxo-2–(2-(1–(4-(2-oxo-2H-chromene-6-sulfonamido)phenyl)ethylidene) hydrazinyl)-4,5-dihydrothiazol-5-yl)acetic acid (15c)

White crystals, mp 193–194 °C, yield (40%). ^1^H NMR (400 MHz, CDCl_3_) *δ ppm*: 2.43 (s, 3H, CH_3_), 2.86 (t, 1H, *J* = 6.4 Hz CH thiazolidinone), 3.04 (d, 2H, *J* = 12.8 Hz CH_2_), 6.58 (d, 1H, *J* = 7.6 Hz, H3 of coumarin), 7.22 (d, 2H, *J* = 6.4 Hz, Ar); 7.53 (d, 1H, *J* = 7.2 Hz, H8 of coumarin), 7.82 (d, 2H, *J* = 6.4 Hz, Ar); 7.95 (dd, 1H, *J* =6.8 and 2 Hz, H7 of coumarin), 8.16 (d, 1H, *J* = 8 Hz, H4 of coumarin), 8.27 (s, 1H, H5 of coumarin), 10.99 (s, 1H, NH), 11.14 (s, 1H, NH), 13.21 (s, 1H, COOH); ^13 ^C NMR (100 MHz, CDCl_3_); *δ ppm*: 16.36 (CH_3_), 38.04 (CH_2_), 42.56 (CH of thiazolidinone), 118.42, 118.59 (2C), 119.51, 128.29, 129.99 (2C), 130.32 (2C), 132.62, 135.63, 142.37, 143.37 (C4 of coumarin), 152.24 (C=N), 156.56 (C9 of coumarin), 159.51 (C=O of coumarin), 165.86 (S-C=N), 172.48 (C=O of COOH), 196.92 (C=O); Analysis for C_22_H_18_N_4_O_7_S_2_ (514), Calcd.: % C, 51.35; H, 3.53; N, 10.89; O, 21.77; S, 12.46. Found: % C, 51.30; H, 3.51; N, 10.86; O, 21.74; S, 12.44.

## Biological evaluation

### *In vitro* anti-proliferative activity

HepG2 liver cancer, MCF-7 breast cancer and Caco-2 cancer cell lines were obtained from the National Cancer Institute (Cairo, Egypt). Caco-2 cells were grown in DMEM while HepG2 and MCF-7 were grown in RPMI-1640. Media were supplemented with 10% heat-inactivated FBS, 50 units/mL of penicillin and 50 g/mL of streptomycin and maintained at 37 °C in a humidified atmosphere containing 5% CO_2_. The cells were maintained as a “monolayer culture” by serial subculturing. Cytotoxicity was determined using the sulforhodamine B (SRB) method as previously described by Skehan et al.^50^ Exponentially growing cells were collected using 0.25% trypsin-EDTA and seeded in 96-well plates at 1000–2000 cells/well in supplemented DMEM medium. After 24 h, cells were incubated for 72 h with various concentrations of the tested compounds as well as doxorubicin as the reference compound. Following 72 h of treatment, the cells were fixed with 10% trichloroacetic acid for 1 h at 4 °C. Wells were stained for 10 min at room temperature with 0.4% SRB dissolved in 1% acetic acid. The plates were air dried for 24 h, and the dye was solubilized with Tris–HCl for 5 min on a shaker at 1600 rpm. The optical density (OD) of each well was measured spectrophotometrically at 564 nm with an ELISA microplate reader (ChroMate-4300, FL, USA). The IC_50_ values were calculated according to the equation for Boltzmann sigmoidal concentrationeresponse curve using the nonlinear regression models (GraphPad, Prism Version 5). The results reported are means of at least three separate experiments. Significant differences were analyzed by one-way ANOVA wherein the differences were considered to be significant at *p* < .05.

### ELISA immunoassay

The levels of the apoptotic markers (Bax, caspase-3) and the anti-apoptotic marker (Bcl-2) were examined using ELISA colorimetric kits per the manufacturer’s protocol and referring to reported instructions^51^ ^,^ ^52^.

### Cell cycle analysis

The HepG2 cells were treated with compound **13a** for 24 h (at IC_50_ concentration), then cells were washed twice with ice-cold phosphate-buffered saline (PBS). Subsequently, the treated cells were collected by centrifugation, fixed in ice-cold 70% (v/v) ethanol, washed with PBS, re-suspended with 0.1 mg/mL RNase, stained with 40 mg/mL PI, and analyzed by flow cytometry using FACS Calibur (Becton Dickinson, BD). The cell cycle distributions were calculated using CellQuest software (Becton Dickinson)^53^.

### Annexin V–FITC apoptosis assay

Phosphatidylserine externalization was measured using Annexin V-FITC/PI apoptosis detection kit (BD Biosciences, San Jose, CA) according to the manufacturer's instructions, as reported earlier^53^. HepG2 cells were treated with **13a** at defined concentrations for 24 h.

## Results and discussion

### Chemistry

**Scheme 1.**
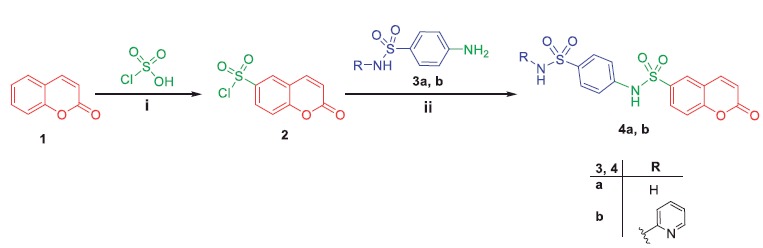
Synthesis of target compounds **4a, b**; *Reagents and conditions*: (**i**) Heating 100 °C, 4 h; (**ii**) EtOH/reflux 12 h.

**Scheme 2.**
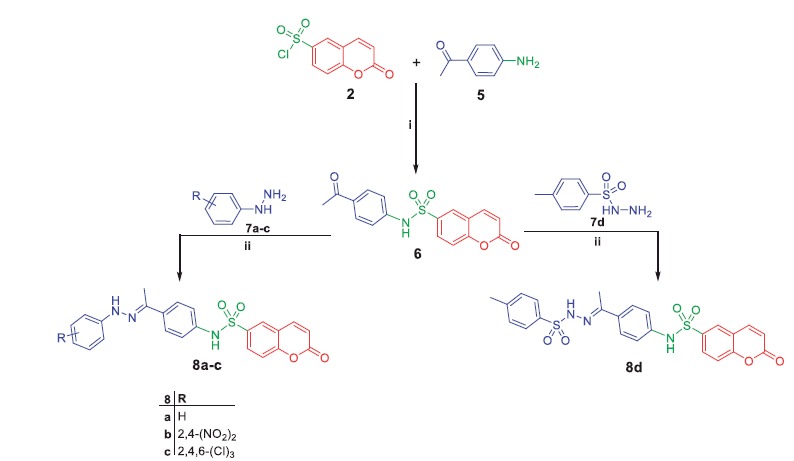
Synthesis of target compounds **8a–d**; *Reagents and conditions*: (**i**) Pyridine/stirring, rt 24h; (**ii**) EtOH/AcOH (catalytic)/reflux 8 h.

**Scheme 3.**
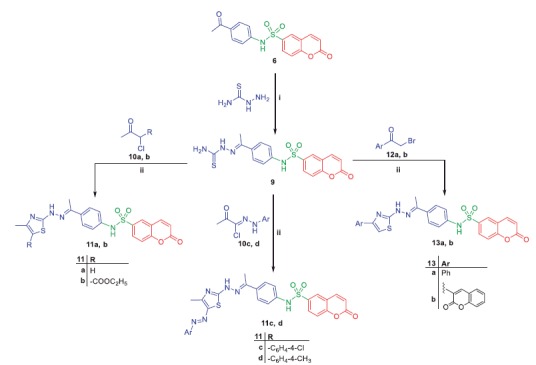
Synthesis of target compounds **11a–d** and **13a, b**; *Reagents and conditions*: (**i**) EtOH/AcOH(catalytic)/reflux 6h (**ii**) Dioxane/TEA (catalytic)/reflux 8h.

**Scheme 4.**
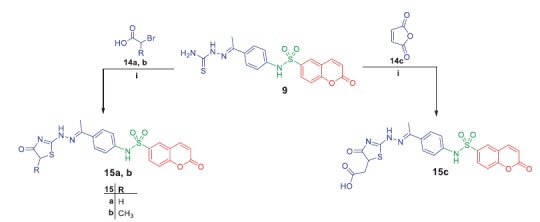
Synthesis of target compounds **15a–c**; *Reagents and conditions*: (**i**) AcOH/anhydrous AcONa/reflux 8h.

The synthetic routes employed to prepare the new target derivatives are depicted in Schemes 1–4. The key intermediate 2-oxo-2*H*- chromene-6-sulfonyl chloride **2** was obtained from a reaction of coumarin **1** with chlorosulfonic acid, which subsequently reacted with sulfanilamide **3a** and sulfapyridine **3 b** in refluxing ethanol to furnish the corresponding target compounds **4a, b**, respectively (Scheme 1).

In Scheme 2, synthesis of *N*-(4-acetylphenyl)-2-oxo-2*H*-chromene-6-sulfonamide **6** was achieved *via* stirring compound **2** with 4-aminoacetophenone **5** in dichloromethane at room temperature. The later was refluxed with phenylhydrazine **7a**, 2,4-dinitrophenylhydrazine **7b**, 2,4,6-trichlorophenylhydrazine **7c** and *p-*toluenesulfonyl hydrazide **7d** in absolute ethanol to afford target coumarins **8a–d**.

Refluxing of compound **6** with thiosemacarbazide in ethanol and catalytic amount of acetic acid yielded the key intermediate **9**, which utilized for preparation of diverse derivatives. Treatment of intermediate **9** with the appropriate α-halocarbonyl compounds **10a–d** in refluxing dioxane furnished the corresponding thiazole derivatives **11a–d**. Additionally, refluxing of **9** with phenacyl bromide **12a** and coumarin-3-acetylbromide **12b** in dioxane afforded the corresponding thiazoles **13a, b**, respectively (Scheme 3). Moreover, treatment of intermediate **9** with each of bromoacetic acid **14a**, 2-bromopropanoic acid **14b** and maleic anhydride **14c** in acetic acid in the presence of anhydrous sodium acetate afforded the target thiazolidinone derivatives **15a–c**, respectively (Scheme 4).

Postulated structures of the newly synthesized coumarin sulfonamides were in full agreement with their spectral and elemental analyses data (Supplementary material).

## Biological evaluation

### *In vitro* anti-proliferative activity

All the newly synthesized target coumarin sulfonamides were evaluated for their *in vitro* anti-proliferative activity against three human tumor cancer cell lines, HepG2 hepatocellular carcinoma, MCF-7 breast cancer, and Caco-2 colon cancer, using SRB colorimetric assay as described by Skehan et al.^50^ Doxorubicin was involved in the experiments as a reference cytotoxic compound. The results were expressed as growth inhibitory concentration (IC_50_) values which represent the compound concentrations required to produce a 50% inhibition of cell growth after 72 h of incubation compared to untreated controls. The results were summarized in Table 1.

**Table 1.** *In vitro* anti-proliferative activity of the target coumarin sulfonamide derivatives against HepG2, MCF-7 and Caco-2 cancer cell lines.

Compound	IC_50_ (µM)^a^		
	HepG2	MCF7	Caco-2
**4a**	35.48 ± 2.93	50.73 ± 4.61	192.69 ± 15.33
**4b**	8.08 ± 0.51	22.95 ± 2.04	66.88 ± 6.07
**6**	25.62 ± 1.36	>200	81.54 ± 6.89
**8a**	26.99 ± 2.01	14.30 ± 1.18	8.53 ± 0.72
**8b**	11.84 ± 1.34	126.08 ± 10.87	89.59 ± 7.26
**8c**	29.80 ± 2.21	>200	76.93 ± 6.59
**8d**	89.91 ± 7.63	>200	16.02 ± 1.32
**9**	163.03 ± 10.22	>200	>200
**11a**	>200	13.86 ± 1.19	10.12 ± 0.90
**11b**	54.31 ± 3.87	63.42 ± 5.69	145.46 ± 8.02
**11c**	72.50 ± 6.33	34.73 ± 2.94	108.41 ± 11.36
**11d**	>200	>200	16.06 ± 1.28
**13a**	3.48 ± 0.28	83.23 ± 6.85	83.43 ± 7.04
**13b**	11.80 ± 1.16	>200	96.64 ± 5.37
**15a**	5.03 ± 0.39	10.95 ± 0.96	158.38 ± 9.39
**15b**	25.07 ± 2.08	10.62 ± 1.35	174.91 ± 12.30
**15c**	7.57 ± 0.66	16.32 ± 1.48	46.06 ± 3.17
Doxorubicin	5.43 ± 0.24	3.18 ± 0.32	4.10 ± 1.37

^a^IC_50_ values are the mean ± SE of three separate experiments.

From the displayed results, it was obvious that several of the prepared coumarin sulfonamides possess excellent to modest growth inhibitory activity towards the tested cancer cell lines. Examinations of the anti-proliferative activity towards HepG2 cells revealed that it is the most sensitive cell line to the influence of the target coumarin sulfonamide derivatives. Compounds **13a** and **15a** emerged as the most active coumarins against HepG2 cells through this study with IC_50_ values of 3.48 ± 0.28 and 5.03 ± 0.39 µM, respectively. They displayed 1.5- and 1.1-fold increased potency than doxorubicin (IC_50_ = 5.43 ± 0.24 µM). Besides, compounds **4b** and **15c** with IC_50_ values of 8.08 ± 0.51 and 7.57 ± 0.66 µM, respectively, displayed excellent activity in comparison to doxorubicin. Also, compounds **8b** and **13b** (IC_50_ = 11.84 ± 1.34 and 11.80 ± 1.16 µM, respectively) exhibited good anti-proliferative activity. Moreover, compounds **6**, **8a**, **8c,** and **15b** had moderate activity with IC_50_ values ranging from 25.07 ± 2.08 to 29.80 ± 2.21 µM.

Concerning activity against MCF-7 cells, compounds **15a** and **15b** were the most active members that displayed potent anti-proliferative activity with IC_50_ values of 10.95 ± 0.96 and 10.62 ± 1.35 µM, respectively, in comparison to the standard drug doxorubicin (IC_50_ = 3.18 ± 0.32 µM). Moreover, compounds **8a**, **11a**, and **15c** were moderately active against MCF-7 cells with IC_50_ values: 14.30 ± 1.18, 13.86 ± 1.19 and 16.32 ± 1.48 µM, respectively. On the other hand, anti-proliferative activity evaluation in Caco-2 cells showed that compound **8a** possessed the best growth inhibitory activity (IC_50_ = 8.53 ± 0.72), with twofold decreased activity than doxorubicin (IC_50_ = 4.10 ± 1.37 µM). In addition, compound **11a** showed good anti-proliferative activity against Caco-2 cancer cell line (IC_50_ = 10.12 ± 0.90). Whilst, both compounds **8d** and **11d** displayed moderate activity against Caco-2 cancer cell line with IC_50_ values: 16.02 ± 1.32 and 16.06 ± 1.28, respectively. These results suggest that diverse functionalities could be incorporated into the coumarin sulfonamide scaffold to obtain promising anti-proliferative activity against different types of cancer cells.

### *In vitro* cytotoxicity towards human normal WI-38 cells

The cell growth inhibitory activity of the most potent compounds **13a** and **15a** was examined towards non-tumorigenic human normal lung fibroblast cell line (WI-38) to investigate the potential safety of the newly synthesized coumarin-6-sulfonamides towards the normal cells. The results were expressed as IC_50_ values, and selectivity index was calculated (Table 2). The tested coumarin-6-sulfonamides (**13a** and **15a**) showed non-significant cytotoxic action with IC_50_ values of 73.20 ± 3.47 and 55.92 ± 0.39 µM, respectively, with good selectivity index range 21 and 11, thereby providing a high safety profile as anticancer agents.

**Table 2.** *In vitro* cytotoxic activity against normal WI-38 cells, and selectivity index of the most active coumarins.

Compound	IC_50_ (µM)		Selectivity index
	WI-38	HepG2	
**13a**	73.20 ± 3.47	3.48 ± 0.28	21
**15a**	55.92 ± 0.39	5.03 ± 0.39	11

### Apoptosis induction in HepG2 cells

Apoptosis induction in cancer cells represents one of the most successful strategies for the development of cancer therapy^54–56^. As displayed above, coumarins **13a** and **15a** emerged as the most active ones towards HepG2 liver cancer cells. Accordingly, we investigated the ability of compounds **13a** and **15a** to provoke apoptosis in HepG2 cells to define the principle mechanism for their anti-proliferative activity.

### Effects on mitochondrial apoptosis pathway proteins Bcl-2 and Bax

The Bcl-2 proteins family is mainly responsible for synchronizing the mitochondrial apoptotic pathway, and classified into two major classes: anti-apoptotic proteins such as Bcl-2 protein and the counteracting pro-apoptotic proteins including Bax protein. In this study, we examined the impact of coumarins **13a** and **15a** on the level of the anti-apoptotic Bcl2 and the level of the pro-apoptotic Bax (Table 3). As shown in Table 3, compound **13a** induced the protein expression of Bax with 16.5 folds of the control while 14.3 folds were recorded for compound **15a**. On the other hand, treatment of HepG2 cells with compounds **13a** and **15a** significantly reduced the expression levels of the anti-apoptotic protein Bcl-2 by 21.4 and 38.6%, respectively, compared to the control.

**Table 3.** Effect of compounds **13a** and **15a** on the active caspase-3 level, and the expression levels of Bcl-2 and Bax in HepG2 cancer cells treated with each compound at its IC_50_ concentration.

Compound	Caspase-3	Bax	Bcl-2
	(ng/mg protein)	(Pg/mg protein)	(ng/mg protein)
**13a**	0.3021 (6.6)^a^	453.3 (16.5)^a^	1.51 (0.21)^a^
**15a**	0.2625 (5.7)^a^	394.3 (14.3)^a^	2.73 (0.39)^a^
Control	0.0457	27.52	7.07

a Numbers given between parentheses are the numbers of folds of control.

### Effects on the levels of active caspase-3 (key executor of apoptosis)

As a key executioner protease, caspase-3 is activated by upstream initiator caspases as caspase-9. Herein, treatment of HepG2 cells with coumarins **13a** and **15a** resulted in a significant elevation in the level of active caspase-3 by about 6.6 and 5.7 folds, respectively, compared to control (Table 3).

### Cell-cycle analysis

**Figure 2.**
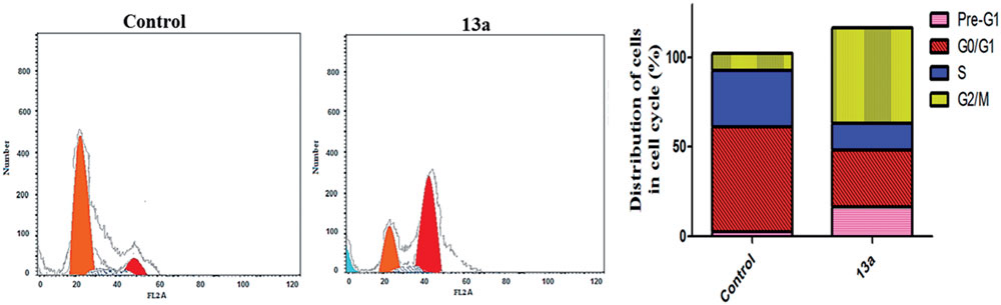
Effect of compound **13a** on the phases of cell cycle of HepG2 cells.

The impact of compound **13a** on cell cycle progression was examined in HepG2 cancer cell line after 24 h of treatment (Figure 2). This impact was illustrated by DNA flow cytometric analysis where HepG2 cells were treated with **13a** at concentration equals to its IC_50_. Figure 2 showed that exposure of HepG2 cells to compound **13a** induced a significant increase in the percentage of cells at Pre-G1 by 6.4-folds, with concurrent significant arrest in the G2-M phase by 5.4-folds compared to control. Alteration of the Pre-G1 phase and arrest of G2-M phase were significant remarks for compound **13a** to induce apoptosis in HepG2 cells.

### Annexin V-FITC apoptosis assay

**Figure 3.**
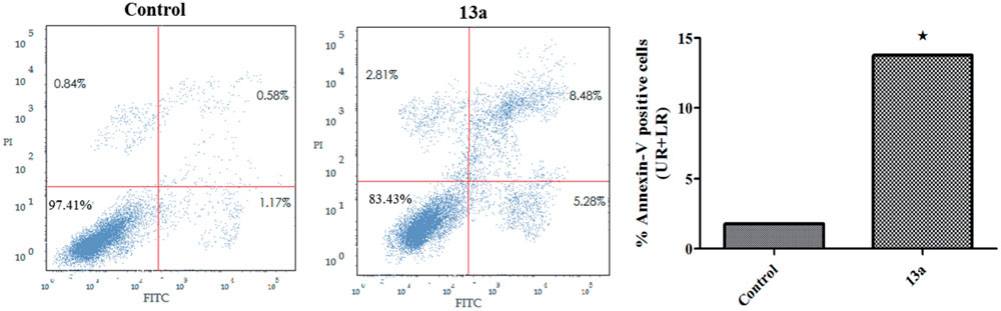
Effect of compound **13a** on the percentage of annexin V-FITC-positive staining in HepG2 cells. The experiments were done in triplicates. The four quadrants identified as: **LL**, viable; **LR**, early apoptotic; **UR**, late apoptotic; **UL**, necrotic. *Significantly different from control at *p* < .05.

The apoptotic effect of coumarin **13a** was further assured by Annexin V-FITC/PI (AV/PI) dual staining assay to investigate the occurrence of phosphatidylserine externalization (Figure 3). Flow cytometric analysis revealed that HepG2 cells treated with **13a** showed a significant increase in the percent of annexin V-FITC positive apoptotic cells (UR + LR) from 1.75% to 13.76% which comprises about 7.9 folds compared to control.

#### 2D QSAR study

##### Development of QSAR models

With the aim to assess the structural basis for the anti-proliferative activity of the newly prepared coumarin sulfonamides (**4a, b**, **6, 8a–d, 9, 11a–d, 13a, b** and **15a**–**c**), 2D-QSAR analysis was carried out. This analysis was run by means of the DS 4.0 software (Discovery Studio 4.0, Accelrys, Co. Ltd)^57^. A set of the newly synthesized compounds (**4a, b**, **6, 8a–d, 9, 11a–d, 13a, b** and **15a–c**) was used as training set with their measured *p*IC_50_ against **HepG2** and **Caco-2** for QSAR modeling, compounds **6**, **8d, 15a** and **15c** were chosen as statistical outliers while building model for **HepG2** whereas, compounds **6**, **9**, and **13a** were chosen for the **Caco-2** model.

“Calculate Molecular Properties” module was used for calculating the molecular properties. Genetic function approximation (GFA) protocol was applied in order to choose the best descriptors that characterize the activity. Multiple linear regression (MLR) protocol was then employed to search for optimal QSAR models with the best statistical validation measures and capable of correlating bioactivity variation across the used training set collection. QSAR model was validated employing leave one-out cross-validation by setting the folds to a number much larger than the number of samples, *r* ^2^ (squared correlation coefficient value) and *r* ^2^ prediction (predictive squared correlation coefficient value), residuals between the predicted and experimental activity of the test set and training set (Table 4).

#### QSAR study results

Equation (1) Represents the best performing QSAR model for the activity against **HepG2**;

(1) $$-logIC_{50}=-0.\text{7926}–\text{1}.\text{598}CHI\_3\_C+0.\text{2775}Dipole\_Y\text{-}\text{3}.\text{1}0\text{2e}-00\text{2}Jurs\_PNSA\_3+0.0\text{4829}Shadow\_YZ$$

**Table 4.** Experimental activities of the synthesized derivatives against the predicted activity according to Equations (1) and (2).

Compound	Caco-2			HepG2		
	Experimental activity	Predicted activity	Residual	Experimental activity	Predicted activity	Residual
	(*p*IC_50_)	(*p*IC_50_)		(*p*IC_50_)	(*p*IC_50_)	
**4a**	−2.2849	−2.2003	−0.0845	−1.5500	−1.5052	−0.0447
**4b**	−1.8253	−2.0638	0.2385	−0.9074	−0.9211	0.0137
**6**	—	—	—	—	—	—
**8a**	−0.9309	−0.8882	−0.0427	−1.4312	−1.5247	0.0935
**8b**	−1.9523	−1.9601	0.0078	−1.0734	−1.0444	−0.0290
**8c**	−1.8861	−1.8261	−0.0600	−1.4742	−1.6672	0.1930
**8d**	−1.2047	−1.1348	−0.0699	—	—	—
**9**	—	—	—	−2.2123	−2.1558	−0.0565
**11a**	—	—	—	−2.3118	−2.1523	−0.1595
**11b**	−2.1627	−2.1391	−0.0237	−1.7349	−1.9201	0.1852
**11c**	−2.0351	−2.0812	0.0462	−1.8603	−1.9921	0.1317
**11d**	−1.2058	−1.5517	0.3459	−2.3222	−2.2134	−0.1088
**13a**	−1.9213	−1.8689	−0.0524	−0.5416	−0.6399	0.0983
**13b**	−1.9852	−1.7932	−0.1920	−1.0719	−1.0082	−0.0637
**15a**	−2.1997	−2.1829	−0.0168	—	—	—
**15b**	−2.2428	−2.1671	−0.0757	−1.3992	−1.1459	−0.2533
**15c**	−1.6633	−1.6425	−0.0208	—	—	—

 

Equation (2) Represents the best performing QSAR model for the activity against **Caco-2**;

(2) $$-{\text{logIC}}_{\text{5}0}=-\text{6}.\text{6}0\text{95}-\text{3}.\text{7896}\text{IAC}\_\text{Mean}+0.\text{11713}\text{Dipole}\_\text{X}-0.\text{1615}\text{Shadow}\_\text{Ylength}$$

**Figure 4.**
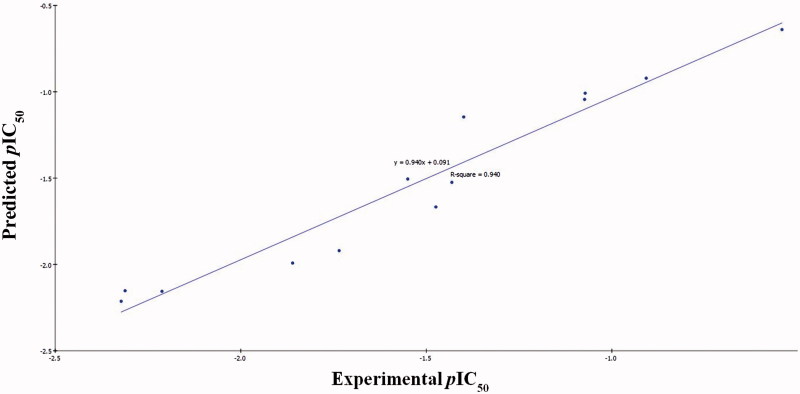
Predicted versus experimental *p*IC_50_ of the tested compounds against HepG2 according to Equation (1) (*r* ^2^ = 0.940).

**Figure 5.**
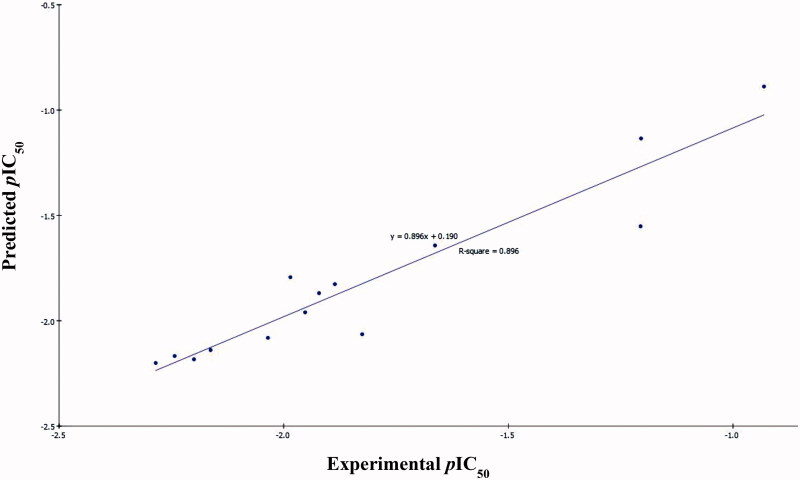
Predicted versus experimental *p*IC_50_ of the tested compounds against Caco-2 according to Equation (2) (*r* ^2^ = 0.896).

According to the former equations these QSAR models were represented graphically by scattering plots of the experimental versus the predicted bioactivity values –log IC_50_ for the training set compounds as shown in Figures 4 and 5.

The two MLR models exhibited good correlation coefficient ***r*^2^** of 0.940 and 0.896, ***r*^2^ (adj)** = 0.910 and 0.865, respectively, ***r*^2^ (pred)** = 0.878 and 0.789, Least-Squared error = 0.0168 and 0.0173 respectively, where *r* ^2^ (adj) is *r* ^2^ adjusted for the number of terms in the model; *r* ^2^ (pred) is the prediction *r* ^2^, equivalent to q^2^ from a leave-1-out cross-validation^58^.

Equations (1) and (2) for **HepG2 and Caco-2** suggested that the anti-proliferative activity of the prepared compounds was positively affected by Dipole, which is a 3D electronic descriptors describing the strength and orientation behavior of a molecule in an electrostatic field. Both the magnitude and the components (X, Y, Z) of the dipole moment are calculated (Debyes). It is estimated by utilizing partial atomic charges and atomic coordinates. Partial atomic charges are computed using Gasteiger if not present. Dipole properties have been correlated to long range ligand-receptor recognition and subsequent binding. The anti-proliferative activity towards **HepG2** was also affected by the high and negative value of Shadow_YZfrac while for **Caco-2** affected by Shadow_Ylength. Shadow indices are a set of geometric descriptors that characterize the shape of the molecules. They are calculated by projecting the model surface on three mutually perpendicular planes: xy, yz, and xz. These descriptors depend not only on conformation, but also on the orientation of the model. In order to calculate them, the models are first rotated to align the principal moments of inertia with the x-, y-, and z-axes. Shadow_YZ is area of the molecular shadow in the yz plane while Shadow_Ylength is indicator for length of molecule in the y dimension^59^.

Equation (1) also showed that the anti-proliferative activity was also affected by jurs descriptors. Jurs descriptors are a group of geometric descriptors that combine both shape and electronic information which may characterize the molecules^60^. In particular, Jurs_PNSA_3, which contributes inversely to the activity, represents the atomic charge weighted negative surface area and calculated by Sum of the product of solvent-accessible surface area × partial charge for all negatively charged atoms. This indicates increasing this value in a molecule could increase reuptake inhibition activity because it is negatively correlated with the activity. Equations (1) and (2) are also affected by topological descriptors which are a special class of descriptors that do not rely on a three-dimensional model. All calculations are derived from the two-dimensional topology of the molecule. As CHI_3_C which is connectivity indices while **IAC_Mean** indicate Graph-Theoretical InfoContent descriptors.

#### QSAR validation

The established QSAR models (1 and 2) were verified by applying; Leave-one-out (LOO) internal validation (***r*^2^** = 0.940 and 0.896, respectively). Cross-validation was also employed where **q** ^2^, which is equivalent to ***r*^2^ (pred),** was 0.878 and 0.789, respectively. In addition, validation was employed by measuring the residuals between the experimental and the predicted activities of the training set listed in Table 4. Interestingly, the predicted activities of the QSAR models were found very close to those experimentally observed, Furthermore, to evaluate the predictive ability of the developed models **HepG2**, compounds **6**, **8d, 15a,** and **15c** were applied as test compounds, where they were not included in model generation, the same was also applied for the **Caco-2** model using compounds **6**, **9**, and **13a** (Table 5).

**Table 5.** External validation for the established QSAR models.

Compound	Caco-2			HepG2		
	Experimental activity	Predicted activity	Residual	Experimental activity	Predicted activity	Residual
	(*p*IC_50_)	(*p*IC_50_)		(*p*IC_50_)	(*p*IC_50_)	
**6**	−1.9114	−1.8158	−0.0956	−1.4086	−0.9815	−0.4271
**8d**	—	—	—	−1.9538	−1.9191	−0.0347
**9**	−2.3424	−1.7150	−0.6275	—	—	—
**13a**	−1.0052	−1.7283	0.7231	—	—	—
**15a**	—	—	—	–0.7016	–0.8816	0.1801
**15c**	—	—	—	–0.8791	–1.1608	0.2817

## Conclusion

In our endeavor to develop potent anti-cancer agents, different sets of coumarin-6-sulfonamide derivatives (**4a, b**, **8a–d**, **11a–d, 13a, b,** and **15a–c**) were synthesized. Anti-proliferative activities of the newly synthesized coumarins was invistigated in three human tumor cancer cell lines, namely, HepG2 hepatocellular carcinoma, MCF-7 breast cancer and Caco-2 colon cancer using sulforhodamine B (SRB) colorimetric assay. Compounds **13a** and **15a** emerged as the most active derivatives towards HepG2 cells (IC_50_ = 3.48 ± 0.28 and 5.03 ± 0.39 µM, respectively), with 1.5- and 1.1-fold increased activity than the reference drug, doxorubicin (IC_50_ = 6.9 ± 2.05 µM), respectively. Besides, compounds **15a** and **15b** were the most active members against MCF-7 cells with IC_50_ values of 10.95 ± 0.96 and 10.62 ± 1.35 µM, respectively. Also, compound **8a** possessed the best growth inhibitory activity against Caco-2 cells (IC_50_ = 8.53 ± 0.72), with 2-fold decreased activity than doxorubicin (IC_50_ = 4.10 ± 1.37 µM). Compounds **13a** and **15a** were able to induce apoptosis in HepG2 cells, as assured by the up-regulation of the Bax and down-regulation of the Bcl-2, besides boosting caspase-3 levels. Besides, compound **13a** induced a significant increase in the percentage of cells at Pre-G1 by 6.4-folds, with concurrent significant arrest in the G2-M phase by 5.4-folds compared to control. Also, **13a** displayed significant increase in the percentage of annexin V-FITC positive apoptotic cells from 1.75% to 13.76%. Moreover, QSAR models were established to explore the structural requirements controlling the anti-proliferative activities.

## Supplementary Material

Supplemental Material
