# Supplementary material for: Novel coumarin-6-sulfonamides as apoptotic anti-proliferative agents: synthesis, in vitro biological evaluation, and QSAR studies
Source: J Enzyme Inhib Med Chem. 2018 Jun 26;33(1):1095–107. doi: 10.1080/14756366.2018.1477137 (PMC6022226; doi:10.1080/14756366.2018.1477137)
Supplement: Supplemental Material [file IENZ_A_1477137_SM5940.pdf]

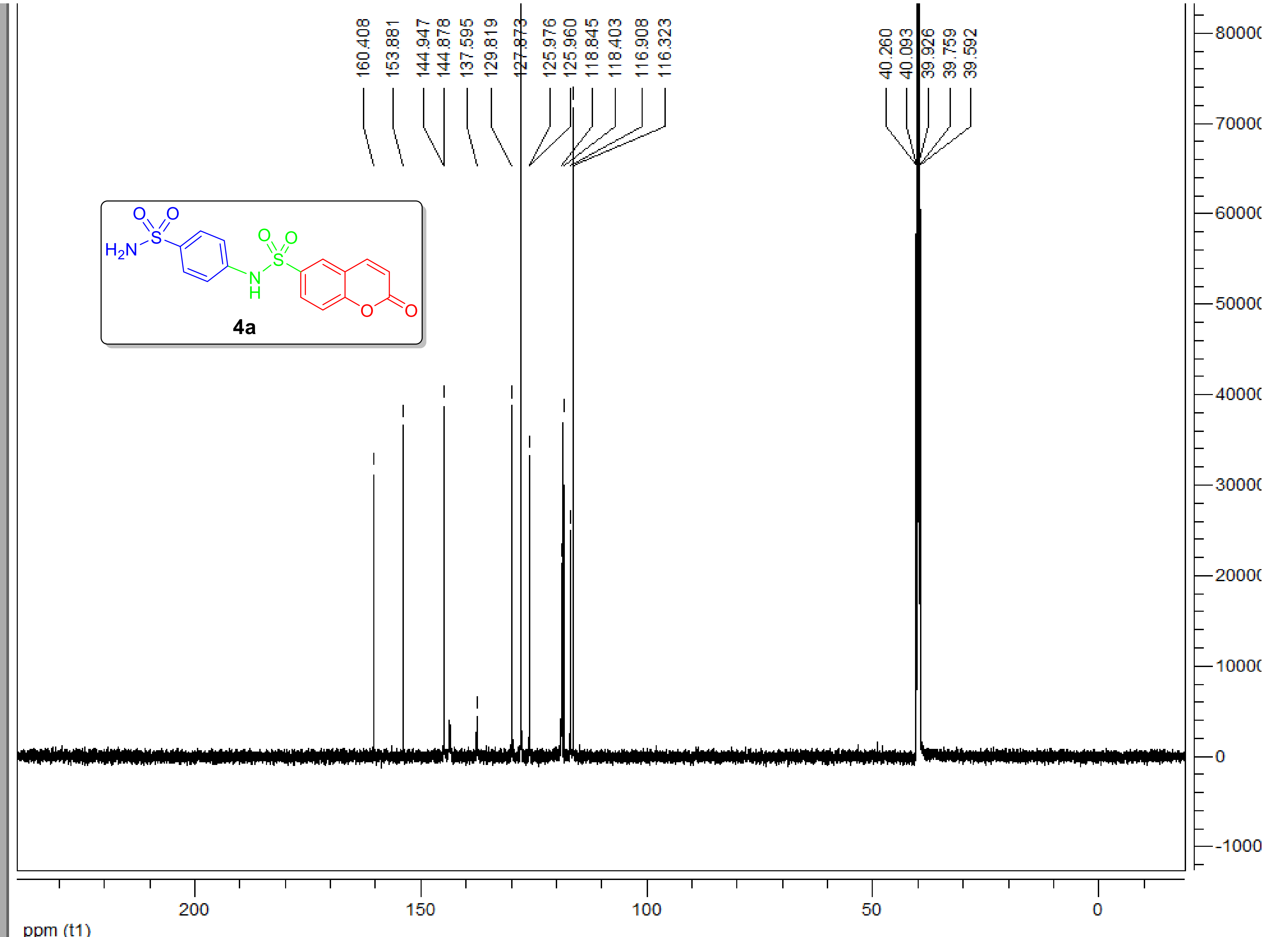

160.408
153.881
144.947
144.878
137.595
129.819
127.873
125.976
125.960
118.845
118.403
116.908
116.323
40.260
40.093
39.926
39.759
39.592
H_2N
S
O
O
N
H
S
O
O
O
O
4a
200
150
100
50
0
ppm (t1)
-1000
0

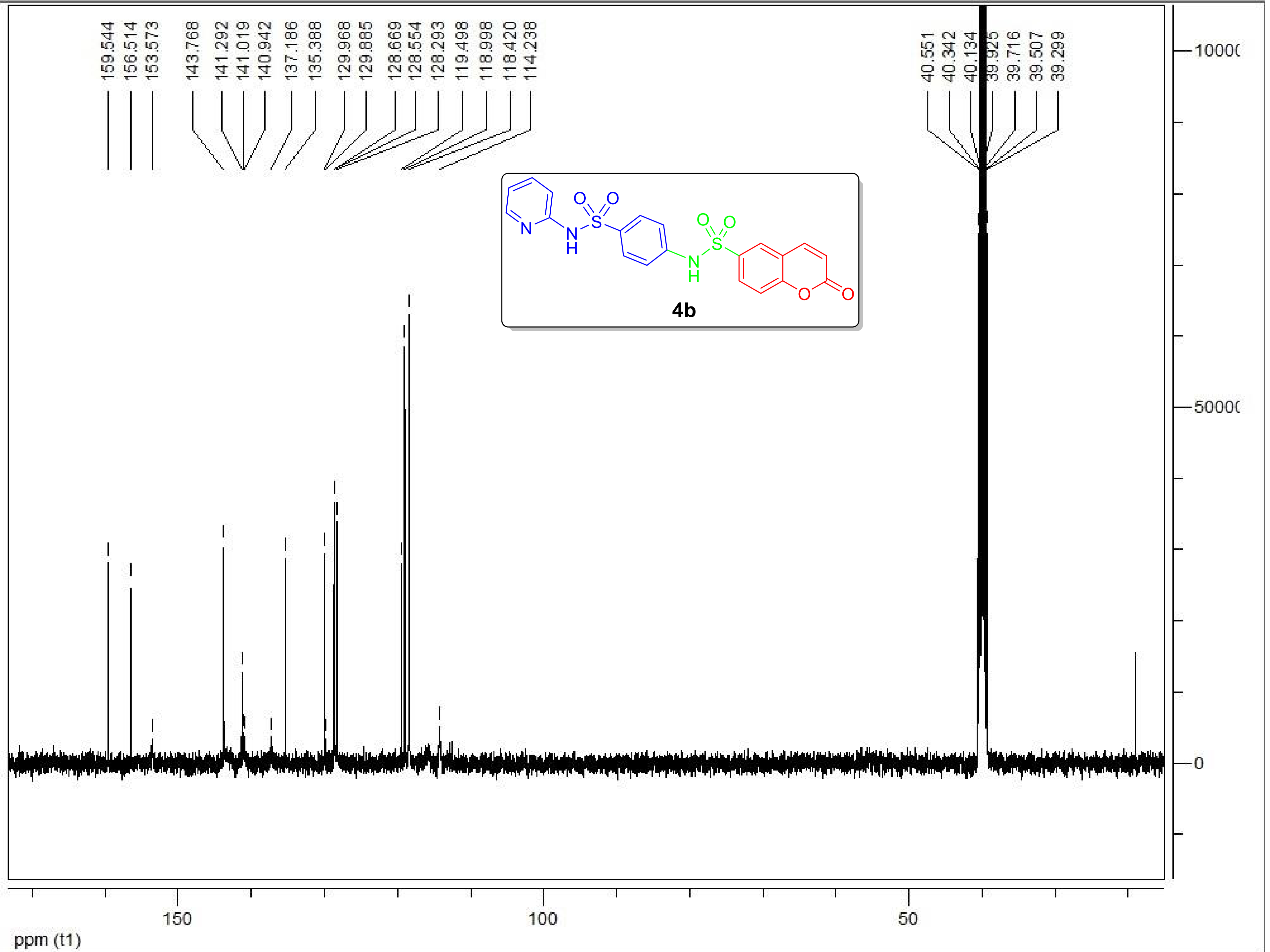
159.544
156.514
153.573
143.768
141.292
141.019
140.942
137.186
135.388
129.968
129.885
128.669
128.554
128.293
119.498
118.998
118.420
114.238
40.551
40.342
40.134
39.925
39.716
39.507
39.299
4b
150
100
50
0
ppm (t1)

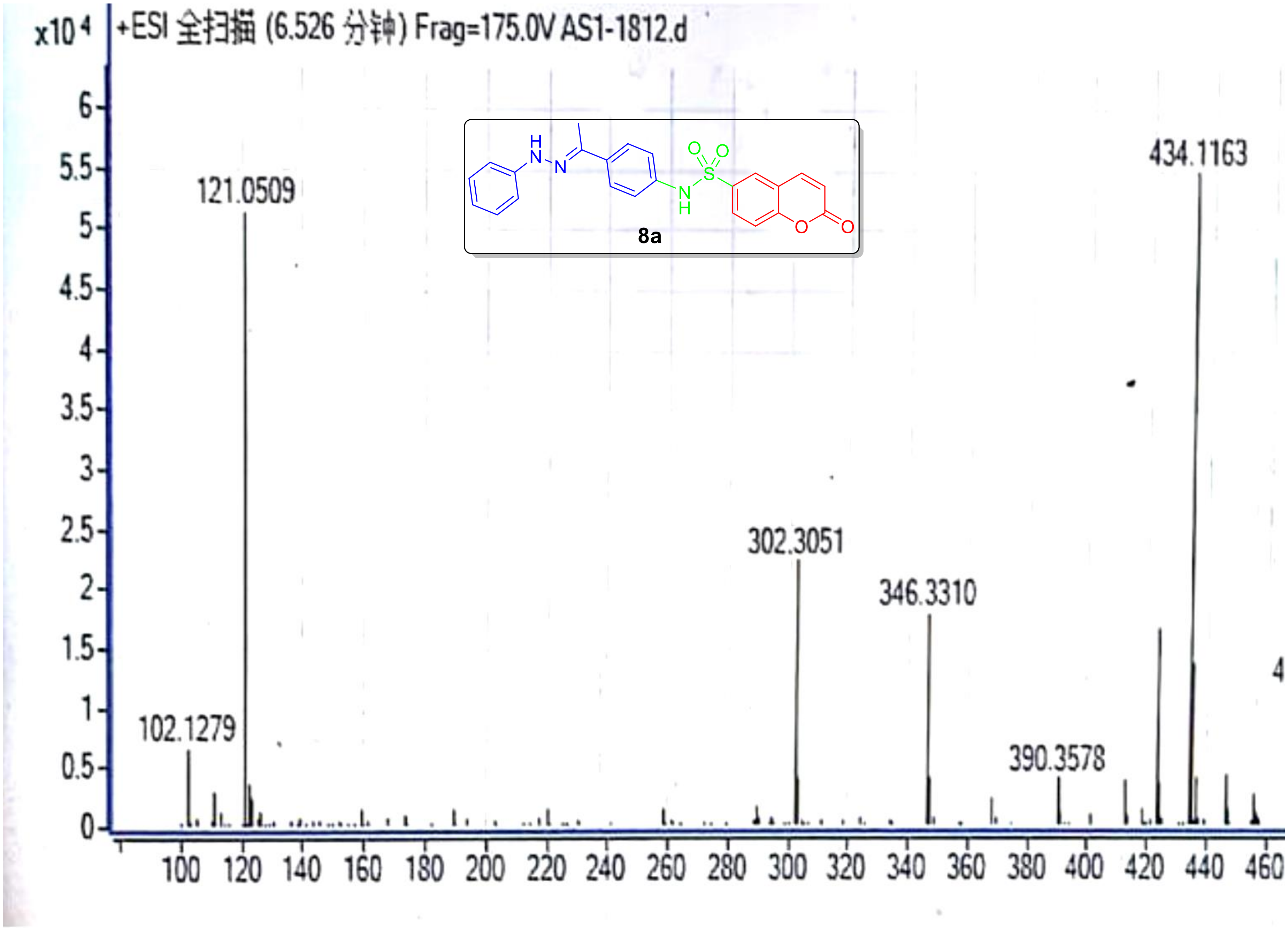
x10 4
+ESI 全扫描 (6.526 分钟) Frag=175.0V AS1-1812.d
121.0509
434.1163
302.3051
346.3310
390.3578
102.1279
8a
H
N
N
O
O
S
N
H
O
O
100 120 140 160 180 200 220 240 260 280 300 320 340 360 380 400 420 440 460
0 0.5 1 1.5 2 2.5 3 3.5 4 4.5 5 5.5 6

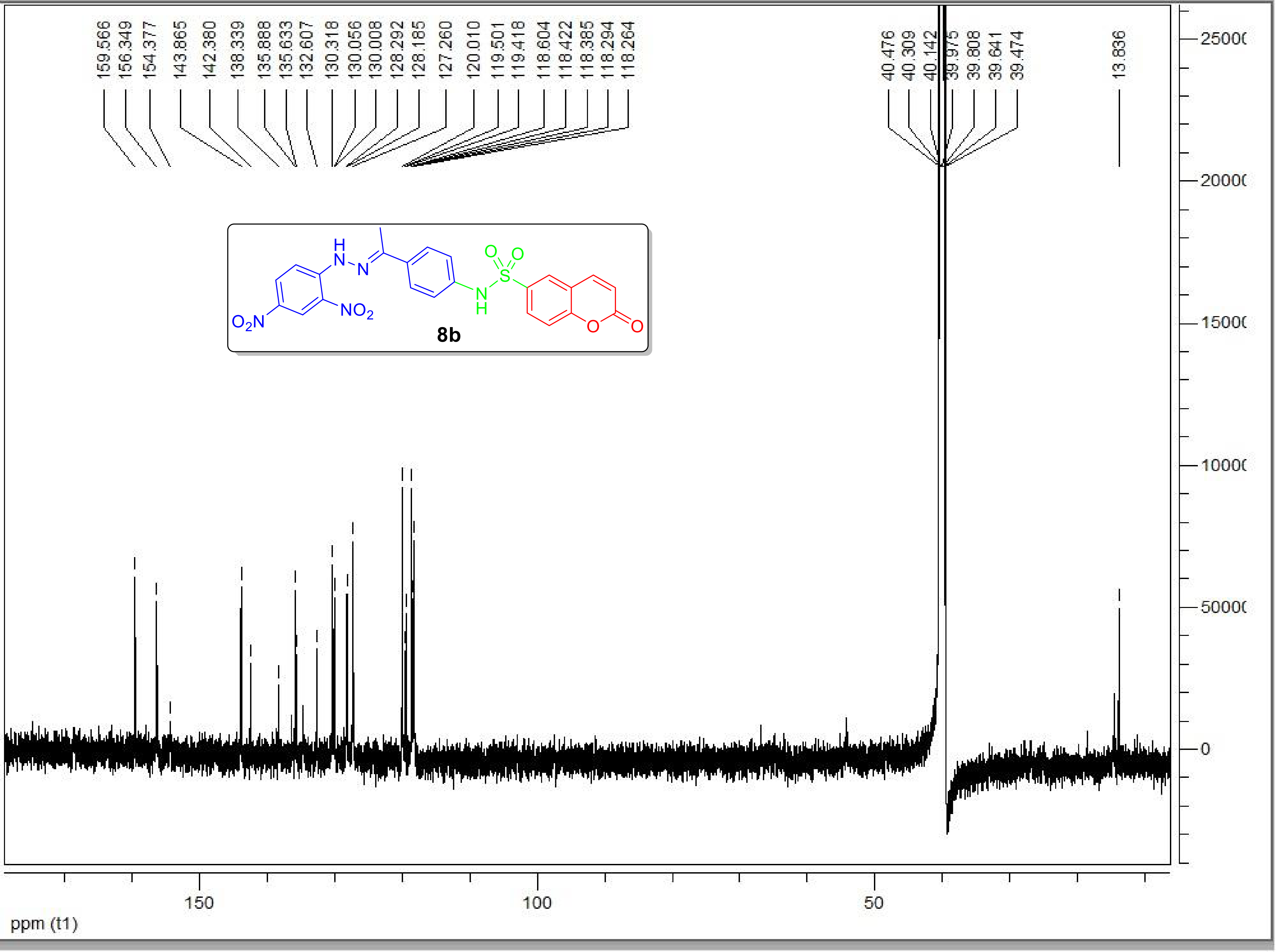
159.566
156.349
154.377
143.865
142.380
138.339
135.888
135.633
132.607
130.318
130.056
130.008
128.292
128.185
127.260
120.010
119.501
119.418
118.604
118.422
118.385
118.294
118.264
40.476
40.309
40.142
39.975
39.808
39.641
39.474
13.836
H
N
N
O
O
S
N
H
O
O
O₂N
NO₂
8b
150
100
50
ppm (t1)
0
50000
10000
15000
20000
25000

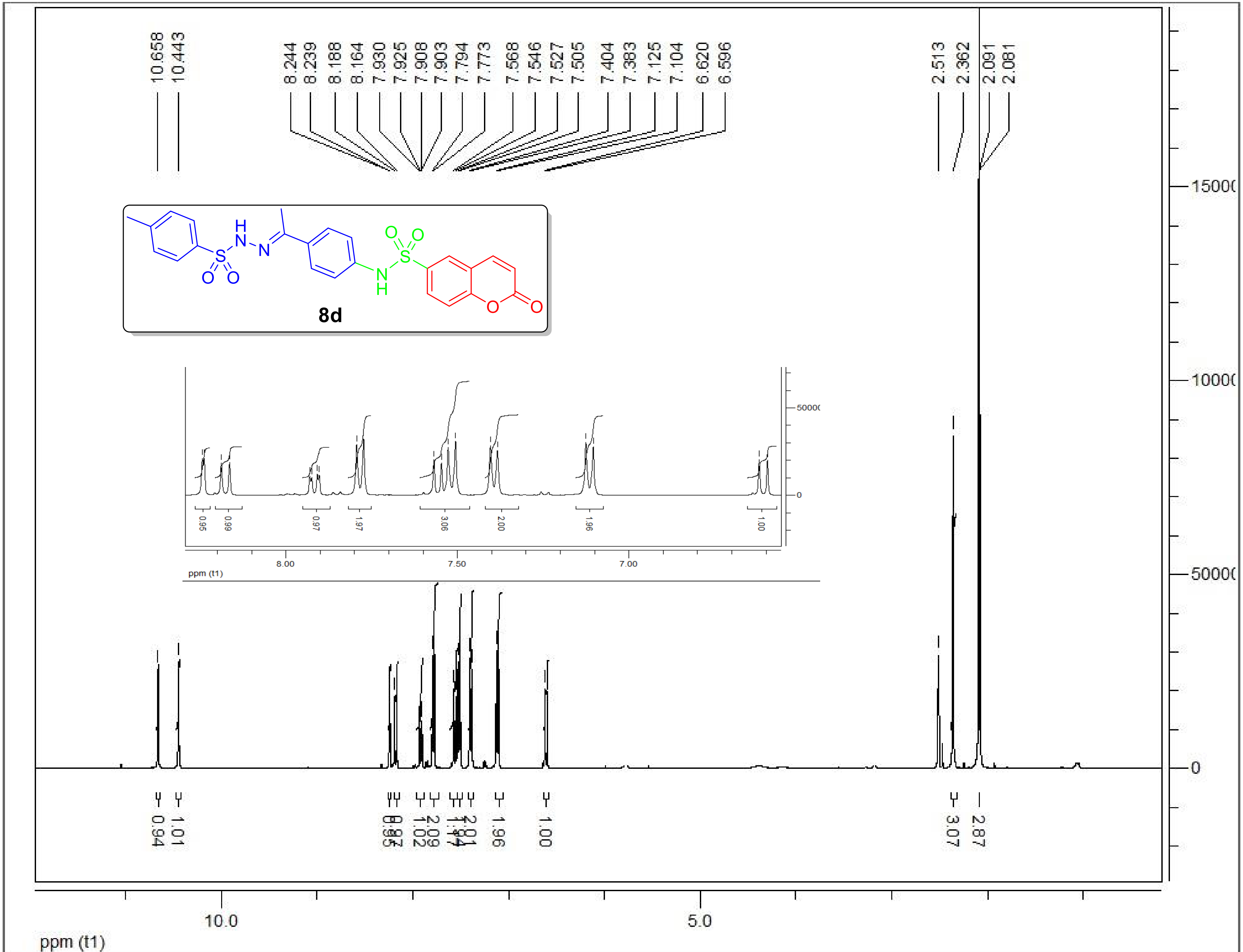

10.658
10.443
8.244
8.239
8.188
8.164
7.930
7.925
7.908
7.903
7.794
7.773
7.568
7.546
7.527
7.505
7.404
7.383
7.125
7.104
6.620
6.596
2.513
2.362
2.091
2.081
8d
0.95
0.99
0.97
1.97
3.06
2.00
1.96
1.00
8.00
7.50
7.00
ppm (t1)
0.94
1.01
1.02
2.09
1.96
1.00
3.07
2.87
15000
10000
50000
0
10.0
5.0
ppm (t1)

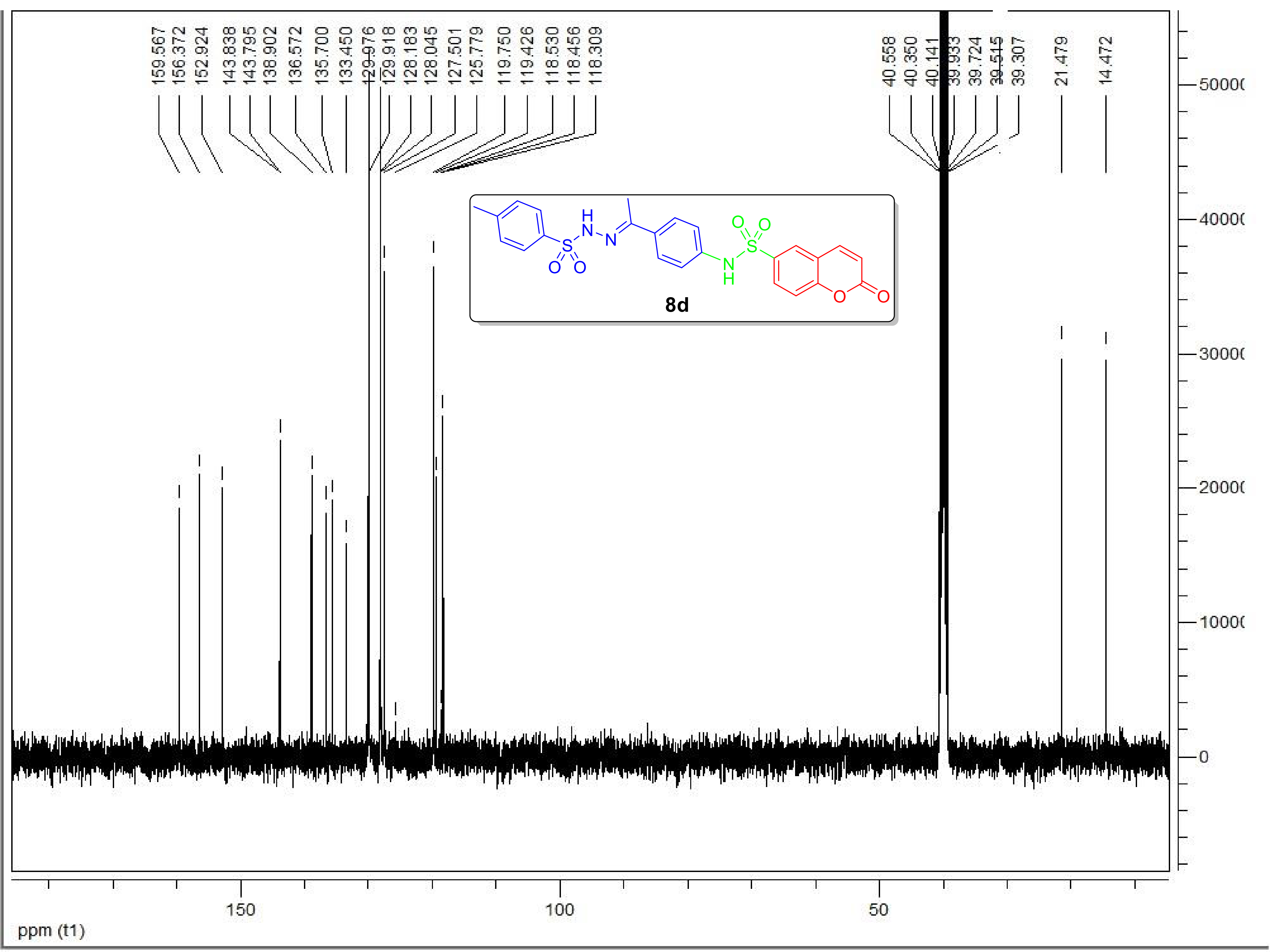
159.567
156.372
152.924
143.838
143.795
138.902
136.572
135.700
133.450
129.976
129.918
128.183
128.045
127.501
125.779
119.750
119.426
118.530
118.456
118.309
40.558
40.350
40.141
39.933
39.724
39.515
39.307
21.479
14.472
8d
50000
40000
30000
20000
10000
0
150
100
50
ppm (t1)

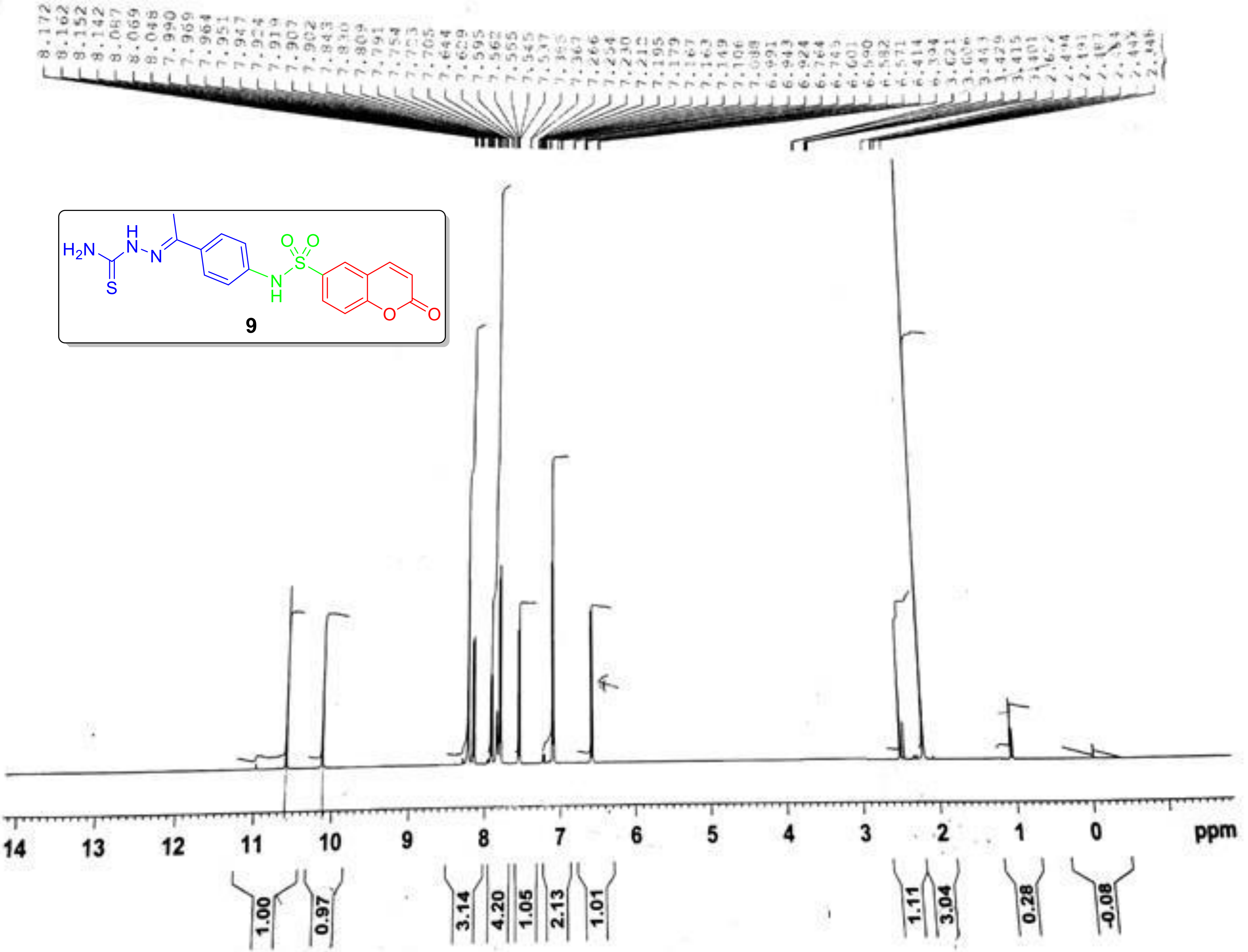
9
14
13
12
11
10
9
8
7
6
5
4
3
2
1
0
ppm
1.00
0.97
3.14
4.20
1.05
2.13
1.01
1.11
3.04
0.28
-0.08

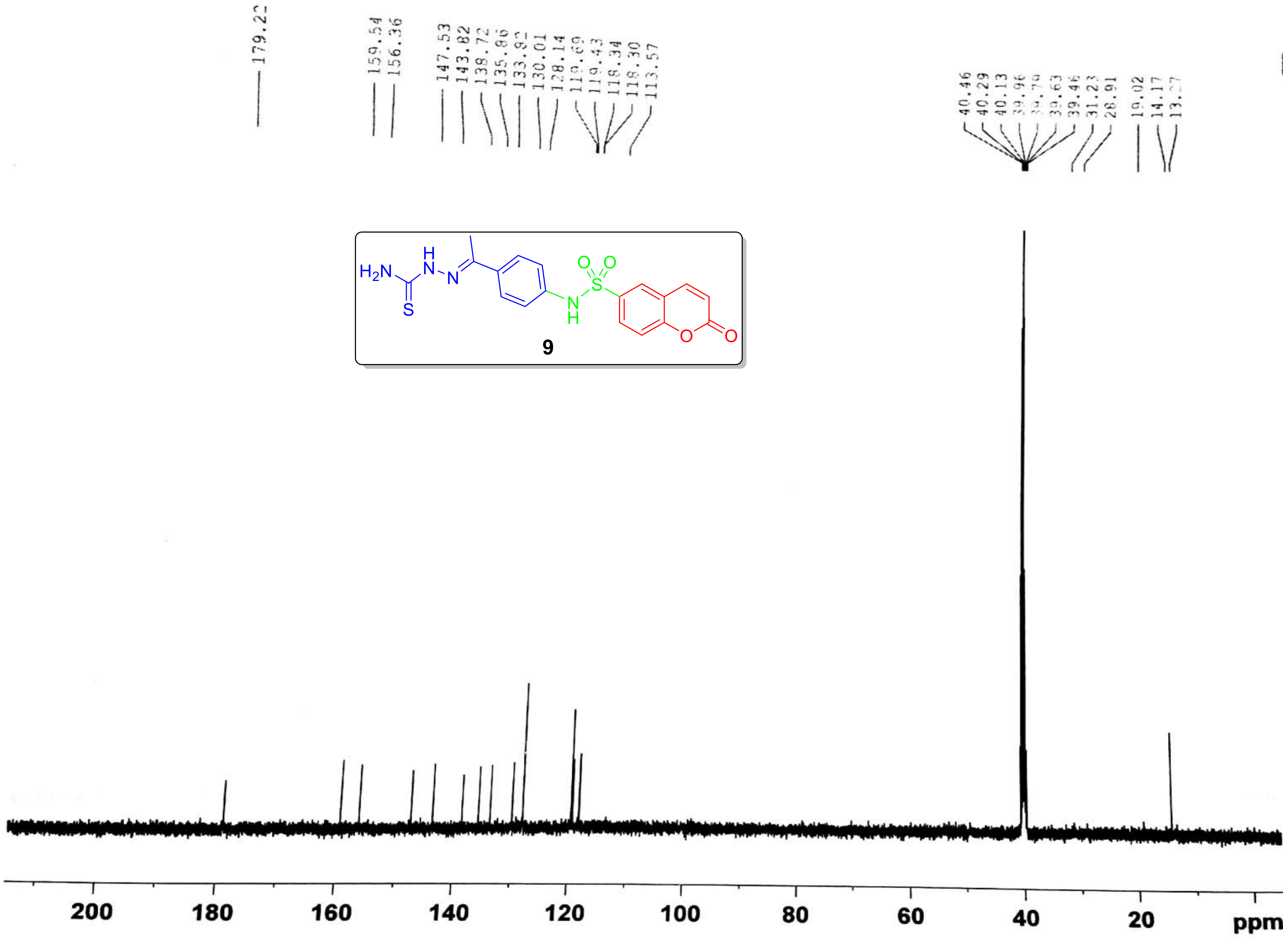
179.22
159.54
156.36
147.53
143.82
138.72
135.86
133.92
130.01
128.14
119.43
118.34
118.30
113.57
40.46
40.29
40.13
39.96
39.63
39.46
31.23
28.91
19.02
14.17
H2N
H
N
N
S
O
O
S
N
H
O
O
9
200
180
160
140
120
100
80
60
40
20
ppm

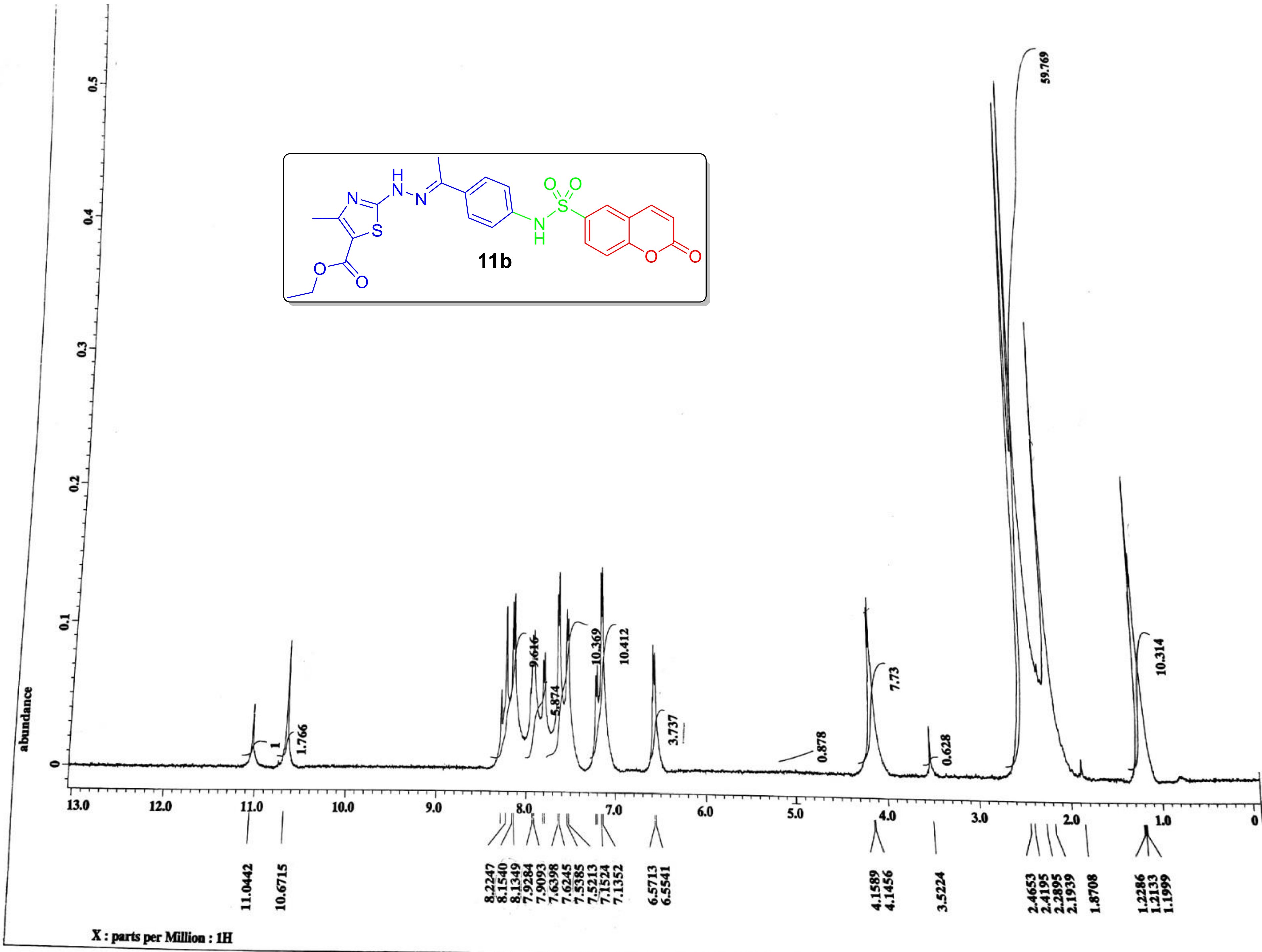
11b
abundance
0
0.1
0.2
0.3
0.4
0.5
59.769
1
1.766
9.616
5.874
10.369
10.412
3.737
0.878
7.73
0.628
10.314
13.0
12.0
11.0
10.0
9.0
8.0
7.0
6.0
5.0
4.0
3.0
2.0
1.0
0
11.0442
10.6715
8.2247
8.1540
8.1349
7.9284
7.9093
7.6398
7.6245
7.5385
7.5213
7.1524
7.1352
6.5713
6.5541
4.1589
4.1456
3.5224
2.4653
2.4195
2.2895
2.1939
1.8708
1.2286
1.2133
1.1999
X : parts per Million : 1H

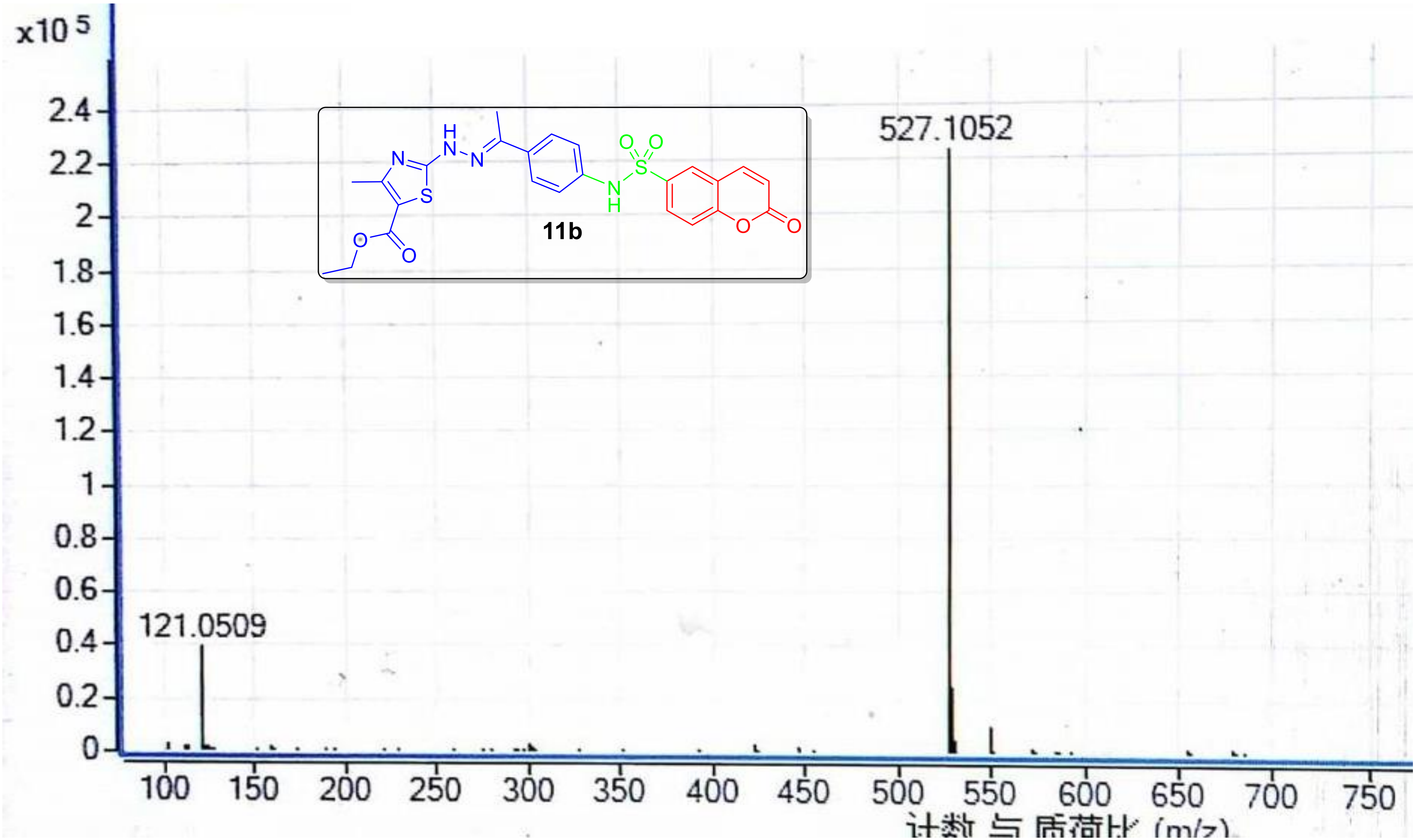
x10 5
2.4
2.2
2
1.8
1.6
1.4
1.2
1
0.8
0.6
0.4
0.2
0
527.1052
121.0509
11b
100
150
200
250
300
350
400
450
500
550
600
650
700
750
计数 与 质荷比 (m/z)

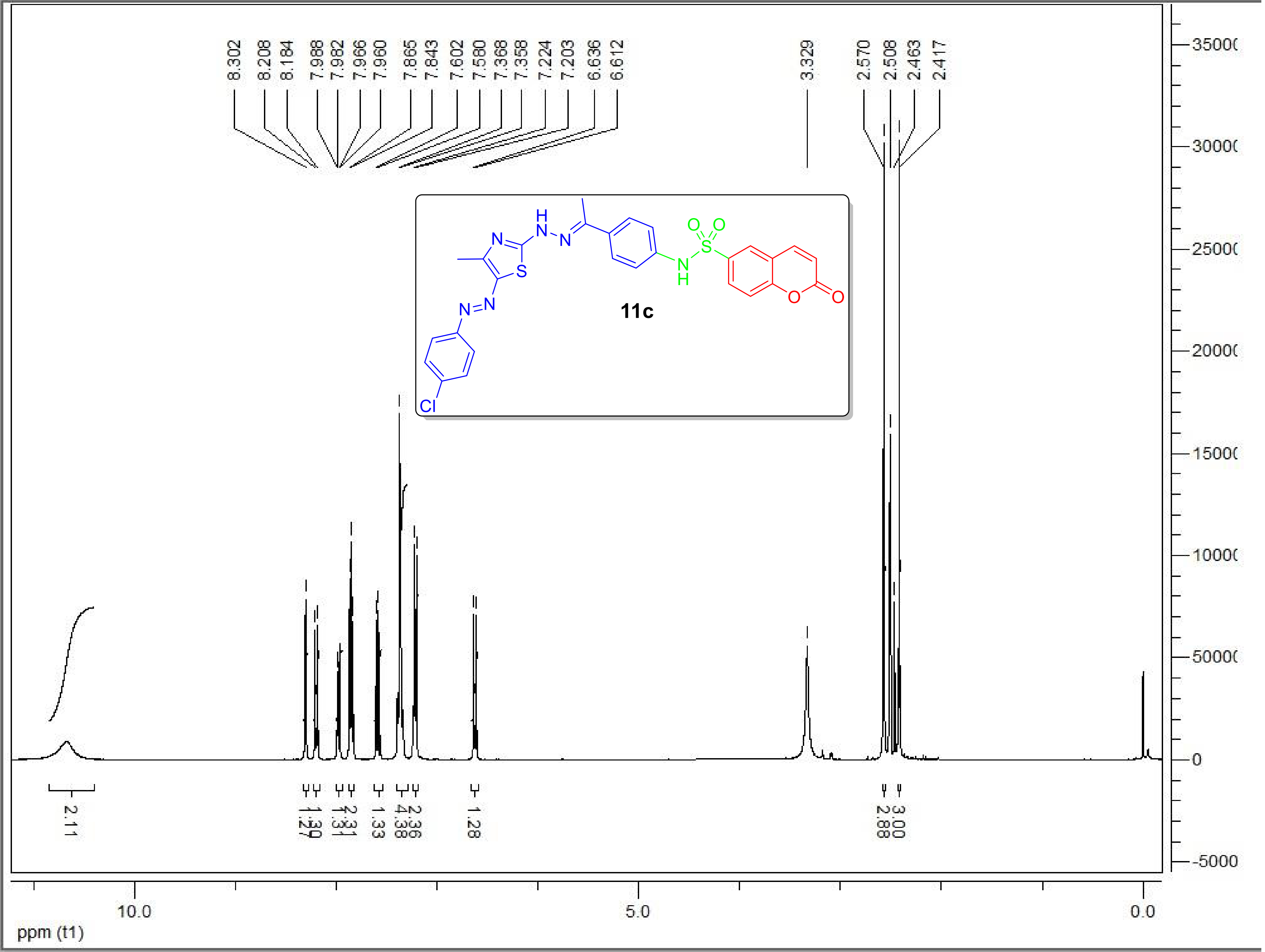

8.302
8.208
8.184
7.988
7.982
7.966
7.960
7.865
7.843
7.602
7.580
7.368
7.358
7.224
7.203
6.636
6.612
3.329
2.570
2.508
2.463
2.417
11c
Cl
2.11
1.29
1.30
1.31
2.31
1.33
4.38
2.36
1.28
2.88
3.00
35000
30000
25000
20000
15000
10000
50000
0
-5000
10.0
5.0
0.0
ppm (t1)

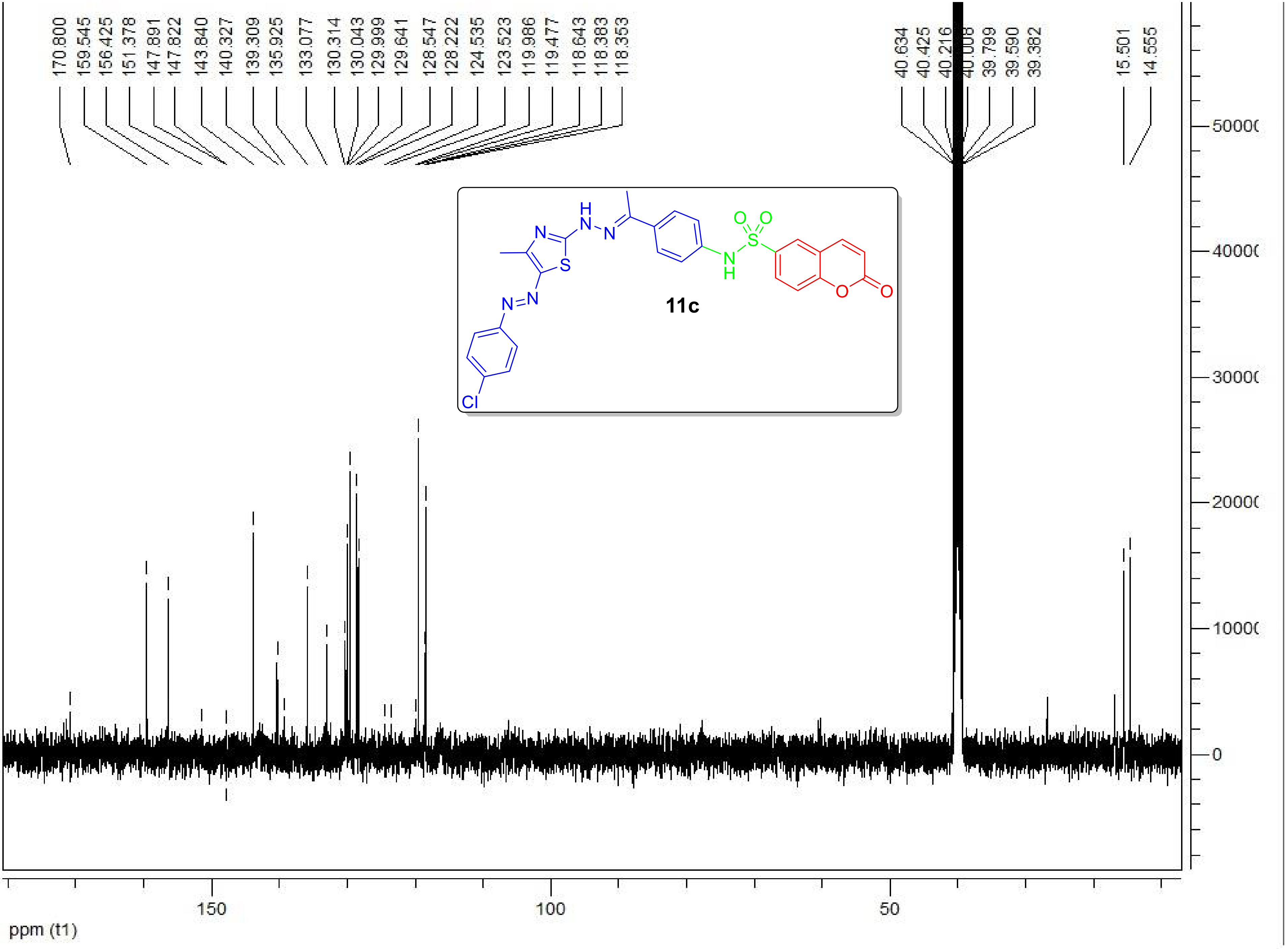
170.800
159.545
156.425
151.378
147.891
147.822
143.840
140.327
139.309
135.925
133.077
130.314
130.043
129.999
129.641
128.547
128.222
124.535
123.523
119.986
119.477
118.643
118.383
118.353
40.634
40.425
40.216
40.008
39.799
39.590
39.382
15.501
14.555
11c
50000
40000
30000
20000
10000
0
150
100
50
ppm (t1)

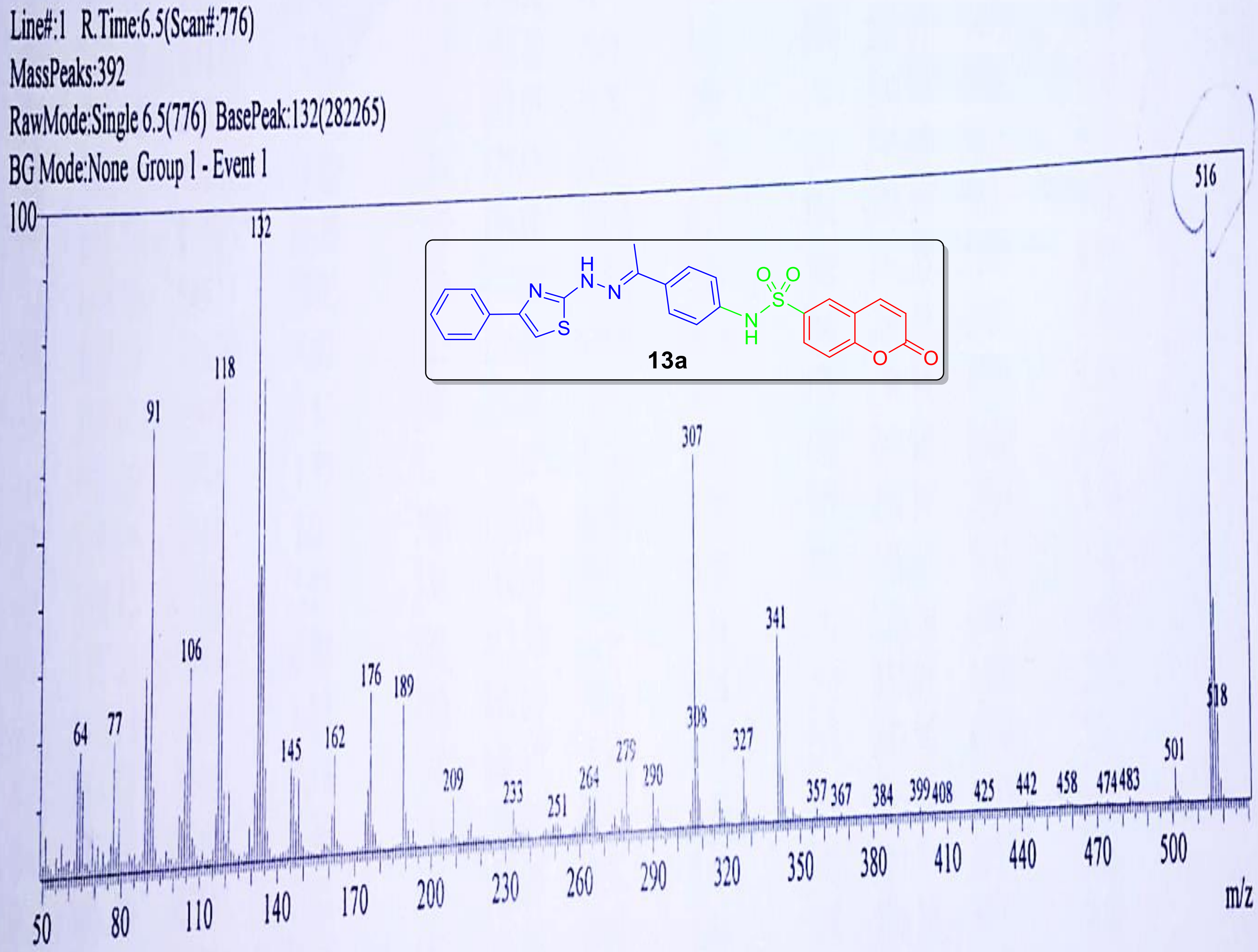

Line#:1 R.Time:6.5(Scan#:776)
MassPeaks:392
RawMode:Single 6.5(776) BasePeak:132(282265)
BG Mode:None Group 1 - Event 1
100
516
132
118
91
307
106
341
176
189
518
64
77
145
162
308
327
501
209
233
251
264
279
290
357 367
384
399 408
425
442
458
474 483
50
80
110
140
170
200
230
260
290
320
350
380
410
440
470
500
m/z
13a

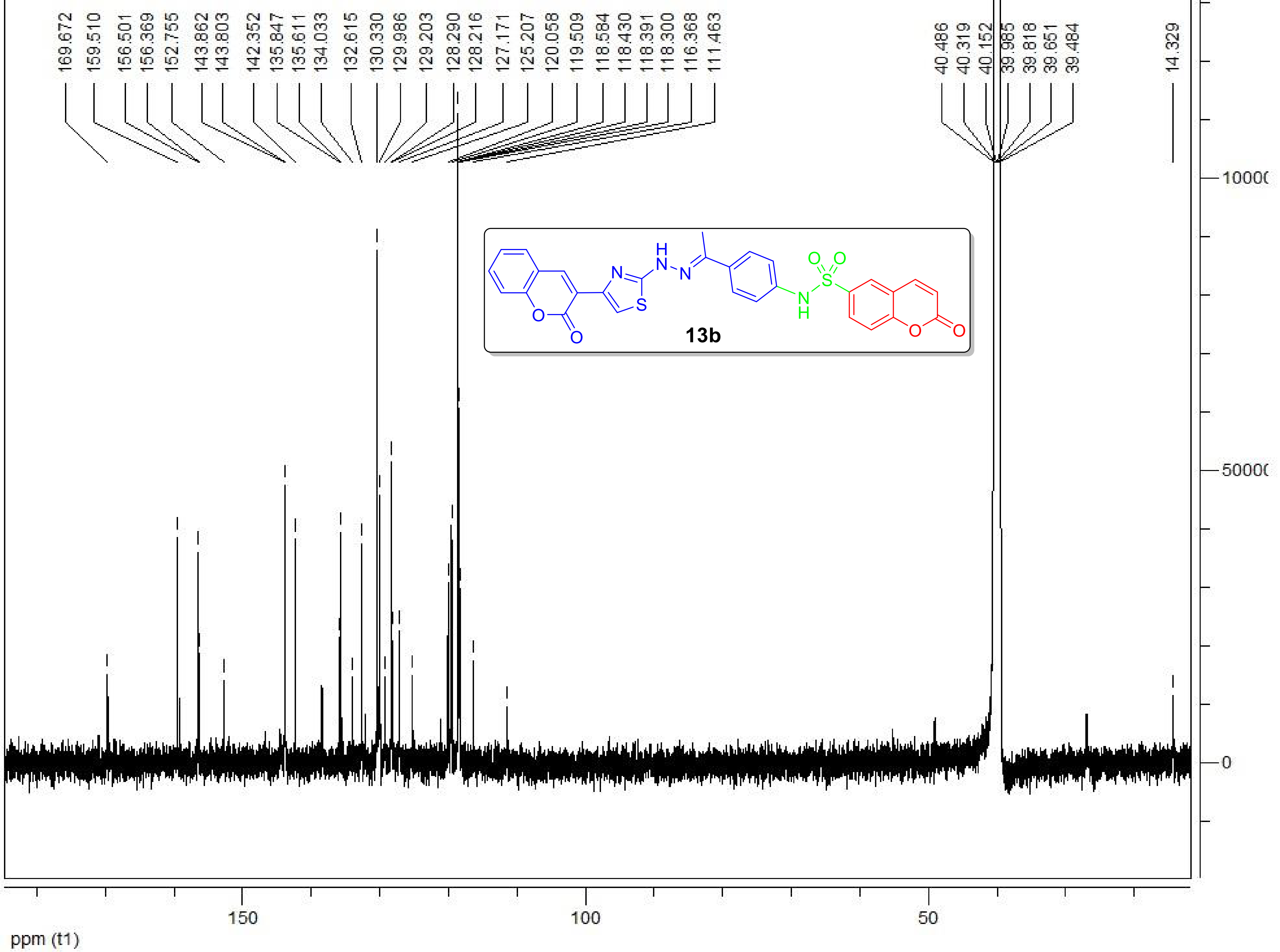
169.672
159.510
156.501
156.369
152.755
143.862
143.803
142.352
135.847
135.611
134.033
132.615
130.330
129.986
129.203
128.290
128.216
127.171
125.207
120.058
119.509
118.584
118.430
118.391
118.300
116.368
111.463
40.486
40.319
40.152
39.985
39.818
39.651
39.484
14.329
13b
10000
50000
0
150
100
50
ppm (t1)
